# Review on Chemical Stability of Lead Halide Perovskite Solar Cells

**DOI:** 10.1007/s40820-023-01046-0

**Published:** 2023-03-31

**Authors:** Jing Zhuang, Jizheng Wang, Feng Yan

**Affiliations:** 1 Department of Applied Physics, The Hong Kong Polytechnic University, Hung Hom, Kowloon, Hong Kong SAR, People’s Republic of China; 2 Beijing National Laboratory for Molecular Sciences, CAS Key Laboratory of Organic Solids, Institute of Chemistry, Chinese Academy of Sciences, Beijing, 100190 People’s Republic of China; 3 University of Chinese Academy of Sciences, Beijing, 100049 People’s Republic of China; 4 Research Institute of Intelligent Wearable Systems, The Hong Kong Polytechnic University, Hung Hom, Kowloon, Hong Kong SAR, 999077 People’s Republic of China

**Keywords:** Perovskite solar cells, Chemical reactions, Defects, Degradation, Device stability

## Abstract

Lead halide perovskite solar cells (PSCs) have become a promising next-generation photovoltaic technology due to their skyrocketed power conversion efficiency. However, the device stability issues may restrict their commercial applications, which are dominated by various chemical reactions of perovskite layers. Hence, a comprehensive illustration on the stability of perovskite films in PSCs is urgently needed. In this review article, chemical reactions of perovskite films under different environmental conditions (e.g., moisture, oxygen, light) and with charge transfer materials and metal electrodes are systematically elucidated. Effective strategies for suppressing the degradation reactions of perovskites, such as buffer layer introduction and additives engineering, are specified. Finally, conclusions and outlooks for this field are proposed. The comprehensive review will provide a guideline on the material engineering and device design for PSCs.

## Highlights

- A comprehensive review is presented on the chemical reactions of perovskite films under different environmental conditions and with charge transfer materials and metal electrodes in perovskite solar cells.
- The influence of chemical reactions on device stability is elucidated.
- Effective strategies for suppressing the degradation reactions are specified.

## Graphical abstract

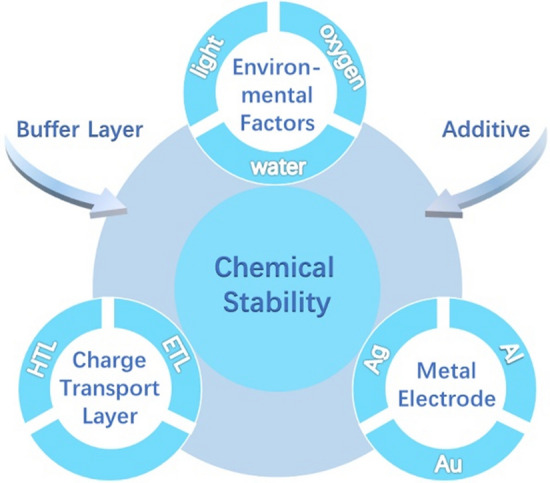

## Introduction

**Fig. 1:**
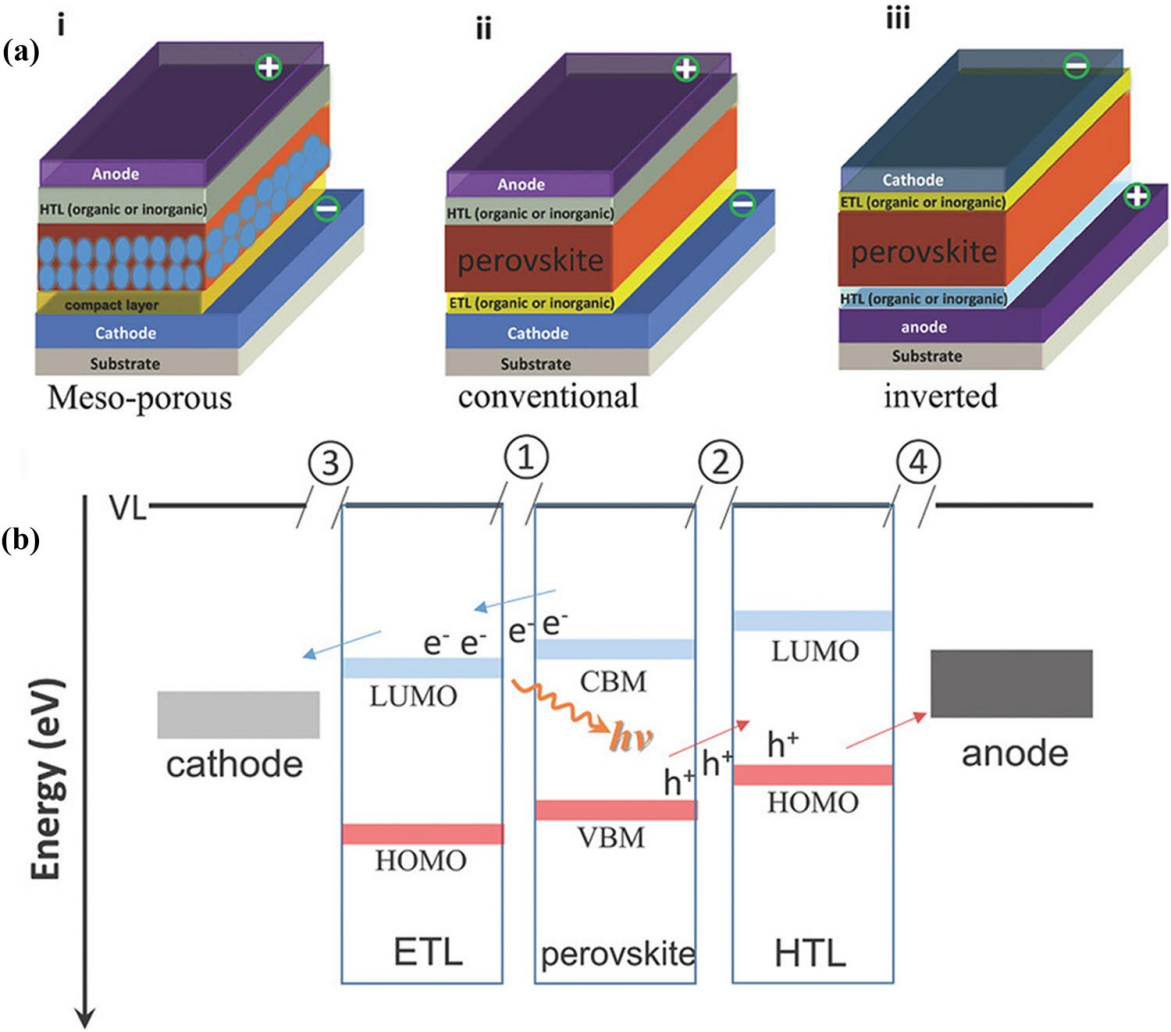
**a** Basic structures of PSCs: (i) mesoporous structure with cathode/compact layer (TiO_2_)/mesoporous layer (TiO_2_ or Al_2_O_3_)/perovskite/HTL/anode, (ii) conventional structure with cathode/ETL/perovskite/HTL/anode, and (iii) inverted structure with anode/HTL/ perovskite/ETL/cathode. **b** Schematic representation of the interfaces in a planar-structured PSC. ➀, ➁, ➂, and ➃ represent ETL/perovskite interface, perovsktite/HTL interface, cathode/ETL interface, and HTL/anode interface, respectively. Reproduced with permission from Ref. [12]. Copyright 2018, Wiley-VCH

As one of the sustainable clean energy sources, photovoltaic technology has been developed vigorously in recent decades. Among them, lead halide perovskite solar cells (PSCs) stand out due to their rapidly increasing power conversion efficiency (PCE), and are currently considered as the most encouraging and promising candidate for the next generation photovoltaic technology [1–7]. In a PSC, the active light-harvesting materials are generally metal halide perovskites with a structural formula of ABX_3_ (A: monovalent cation, CH_3_NH_3_^+^, HC(NH_2_)_2_^+^, Cs^+^; B: divalent metal cation, Pb^2+^, Sn^2+^; X: halide anion, I^−^, Br^−^, Cl^−^) [8–11], which are responsible for converting the incident sunlight into free carriers in the devices, therefore playing a crucial role in the conversion of energy. The extraction of free carriers depends on the electron/hole transport layers (ETL/HTL) that sandwich the perovskite layer and transport charge carriers to corresponding electrodes. Therefore, typical configurations of PSCs involve conductive glass/ETL/perovskite/HTL/electrode (n-i-p) or conductive glass/ HTL/perovskite/ ETL/electrode (p-i-n), as shown in Fig. 1 [12–16].

PSCs show advantages over commercially available solar cells in terms of low cost and little energy consumption in device fabrication. As the dominating share of the photovoltaic industry, monocrystalline silicon solar cells suffer from high fabrication cost, high-temperature preparation, bulky active layer and long payback time. In contrast, PSCs can be fabricated by solution methods at low temperature with very low fabrication cost and little material consumption [17–23]. GaAs solar cells, which have realized the highest PCE of single-junction solar cells, are only used in small-area markets such as space stations [24], while large-area PSCs (over 60 cm^2^) with PCE over 20.5% have been achieved [25, 26], promising their large-area applications. Other thin film solar cells, such as copper indium diselenide (GIGS), cadmium telluride (CdTe), and quantum dot solar cells, are subject to the slow growth of efficiency [27]. Notably, the certified PCE of PSCs has exceeded 25.7% after one decade study, owing to the excellent photophysical properties of perovskites [5]. Considering the theoretical Shockley–Queisser limit of ~ 31% [28–31], there is still a relatively large space for PCE improvement for PSCs. Despite these advantages, device stability of PSCs is a critical issue to their commercial applications [32–37]. It has been recognized that device stability is closely related to a series of chemical reactions between perovskite and ETL/HTL [5, 38–40], electrodes or the environment issues (e.g., moisture, oxygen, light) [41–46].

The chemical reactions of lead halide perovskite have significant impacts on interfacial defects [47, 48], charge transport/extraction [49], and thus photovoltaic performance and device stability of PSCs [50–55]. Because of the relatively active chemical properties, perovskites may react with oxygen [56–59], water [60–63], Lewis acids and bases [64, 65] and some metals [66–70], at perovskite/charge transport layer interfaces or grain boundaries (GBs). In general, these reactions fall into two categories: 1) the reactions with species in ambient environment, including water, oxygen, and light; 2) the reactions with other materials in the device, including HTL/ETL, metal electrodes, and alternative modifiers. In order to suppress the degradation reactions of perovskites, it’s necessary to reduce the reactivity of perovskites through doping additives or compositional modifications [65, 71–77]. Despite significant efforts in additive engineering, the stability of PSCs still can’t meet the requirements for commercialization [78–81]. Physical isolation, another effective strategy for improving device stability, also attracts enormous attention simultaneously [82–89]. Some specific materials can serve as buffer layers to reduce the detrimental interfacial reactions. For example, the modified ZnO isolation layer with good electrical conductivity between the Ag electrode and ETL can effectively suppress the reaction between perovskite and Ag [90]. The hydrophobic diketopyrrolopyrrole-based polymers are deposited on perovskites surfaces to prevent the perovskites from being invaded by water [91]. In conclusion, understanding the chemical reactions at the interfaces can help researchers find feasible approaches to prevent the degradation of lead halide perovskites and improve the stability of PSCs.

In this review, we describe a variety of interfacial chemical reactions under different conditions, and the reactions mechanisms are also summarized. We retrospectively examined the established understanding of how the interfacial reactions affect the defects/traps formation, non-radiative recombination, ion migrations, and eventually device stability. A relatively clear relationship between device stability and interfacial chemical reactions is revealed in this part. According to different chemical reactions mechanism, we also review some available strategies for improving the chemical stability of perovskites. Finally, potential suggestions on reducing or avoiding the detrimental interfacial reactions in device fabrication and commercialization are proposed.

## Interfacial Chemical Reactions

### Environmental Factors

Although encapsulated lead halide PSCs can insulate air, the challenge of achieving an ideal encapsulation effect makes it necessary to explore how environmental factors affect the chemical stability of PSCs. Both pure MAPbI_3_ and FAPbI_3_ are very sensitive to air due to their intrinsic instability, which can be demonstrated by their color changes from black to yellow in a few minutes, especially in high-humidity environments. Studies have shown that water and oxygen in the air can react with perovskites through different pathways. In addition, ambient light can induce the decomposition of perovskites, which will be discussed in this part.

#### Water-Induced Reactions

**Fig. 2:**
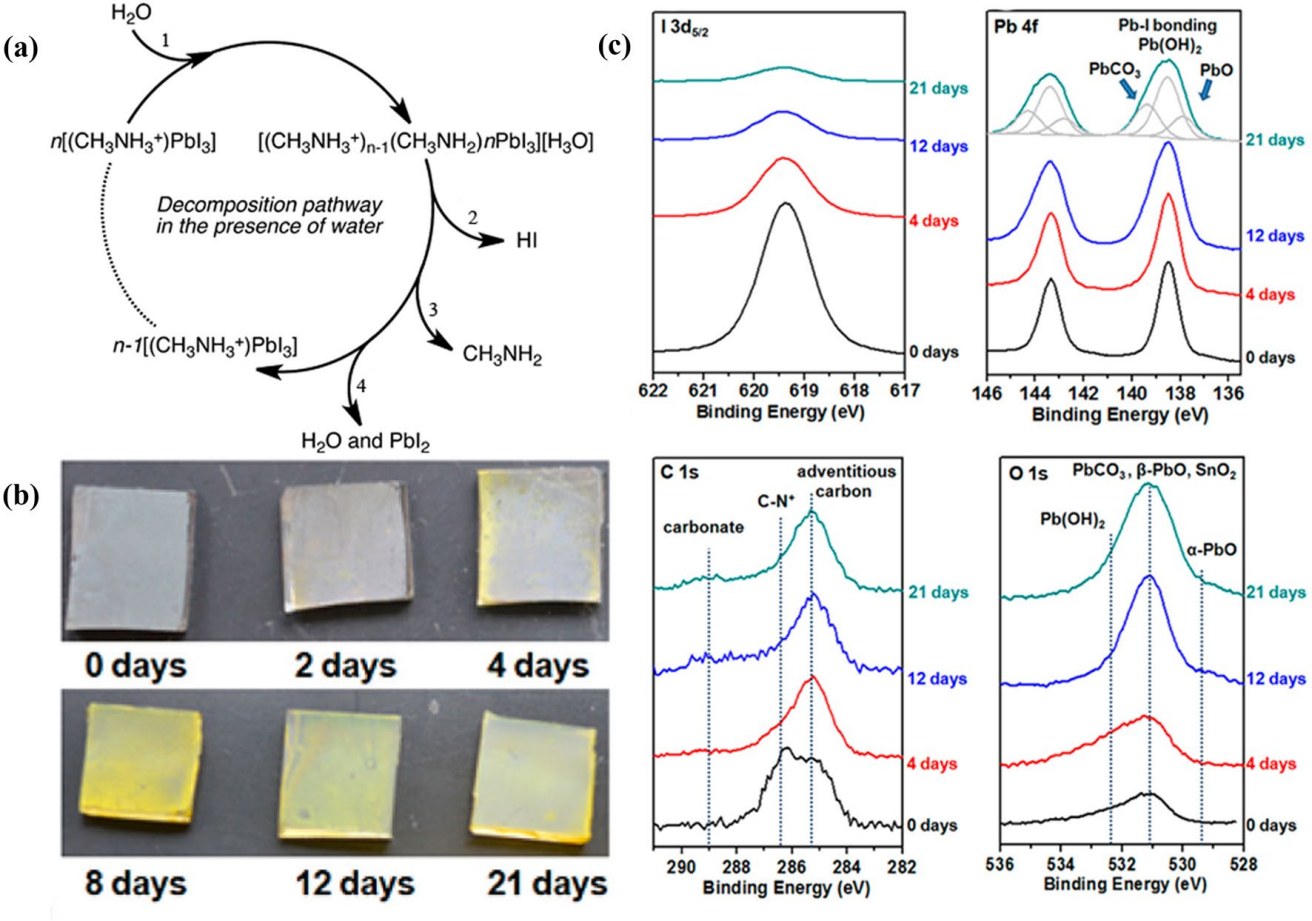
**a** Possible decomposition pathway of hybrid halide perovskites in the presence of water. A water molecule, 1, is required to initiate the process with the decomposition being driven by the phase changes of both hydrogen iodide, (2, soluble in water) and the methylammonia (3, volatile and soluble in water). This pathway results in the formation of a yellow solid, which corresponds to the experimentally observed PbI_2_, 4. Reproduced with permission from Ref. [48]. Copyright 2014, American Chemical Society. **b** Photographs of CH_3_NH_3_PbI_3_ films deposited on FTO and stored under ambient conditions for several days. **c** Evolutions of photoelectron spectra of I 3*d*5/2, Pb 4*f*, C 1*s* and O 1*s*. **b**,** c** Reproduced with permission from Ref. [97]. Copyright 2016, American Chemical Society. (Color figure online)

Many studies have revealed that the reactions of lead halide perovskites with H_2_O can accelerate the degradation process and deteriorate the chemical stability of PSCs [60, 61, 92]. In order to express the reaction mechanism more concisely, all chemical reactions of perovskite in this review are based on the archetypal MAPbI_3_ unless otherwise stated. Walsh et al*.* proposed the simple acid–base reversible reactions between MAPbI_3_ and H_2_O as follows [48], and a plausible decomposition pathway for MAPbI_3_ is shown in Fig. 2a.

2.1-1 $$ \left[ {(CH_{3} NH_{3} )PbI_{3} } \right]_{n}  + H_{2} O{ \leftrightharpoons }\left[ {(CH_{3} NH_{3} )_{{(n - 1)}} (PbI_{3} )_{n} } \right][H_{3} O^{ + } ] + CH_{3} NH_{2}  \uparrow  $$

2.1-2 $$ \left[ {(CH_{3} NH_{3} )_{{(n - 1)}} (PbI_{3} )_{n} } \right][H_{3} O^{ + } ] \rightleftharpoons HI \uparrow  + PbI_{2}  + \left[ {(CH_{3} NH_{3} )PbI_{3} } \right]_{{(n - 1)}}  + H_{2} O $$

By combining Eqs. 2.1-1 and 2.1-2, the whole degradation process of MAPbI_3_ in the presence of H_2_O can be generalized as follows:

2.1-3 $$ \left( {CH_{3} NH_{3} } \right)PbI_{3} \xrightarrow{{H_{2} O}}HI \uparrow  + PbI_{2}  + CH_{3} NH_{2}  \uparrow   $$

CH_3_NH_2_ and HI both exist in gas phases at room temperature, and thus the continuous release of gas products promotes the reaction to proceed in the forward direction. In consequence, MAPbI_3_ will completely degrade into PbI_2_ once the open system contains water [93].

Wang et al*.* further proposed that the degradation product HI can continue to decompose into H_2_ and I_2_ under the stimulation of ultraviolet (UV) light [94]. The photoreaction process of HI under UV irradiation is illustrated below:

2.1-4 $$ 2HI\xrightarrow{{UV}}H_{2}  \uparrow  + I_{2}  $$

The whole degradation reaction of MAPbI_3_ when H_2_O and UV light coexist is generalized in Eq. 2.1-5 with the combination of Eqs. 2.1-3 and 2.1-4. Therefore, the final solid products of MAPbI_3_ decomposition are PbI_2_ and I_2_.

2.1-5 $$ \left( {CH_{3} NH_{3} } \right)PbI_{3} \xrightarrow{{H_{2} O + UV}}\frac{1}{2}H_{2}  \uparrow  + PbI_{2}  + CH_{3} NH_{2}  \uparrow  + \frac{1}{2}I_{2}    $$

Considering that lead halide perovskites are prone to absorb H_2_O molecule in the air to form a hydrated complex [50, 51, 63], the hydration processes of MAPbI_3_ are displayed in Eqs. 2.1-6 and 2.1-7, respectively. Notably, the decoloring process of black perovskites induced by the generation of monohydrated phase CH_3_NH_3_PbI_3_·H_2_O is reversible [95], and that can be reconverted into MAPbI_3_ again through dehydration process [63]. However, the degradation reactions become irreversible once the dihydrate (CH_3_NH_3_)_4_PbI_6_·2H_2_O forms (Eq. 2.1-7). Here, in the hydration process_,_ the [PbI_6_]^4−^ in the 3D network of MAPbI_3_ decays to a 0D framework of isolated octahedral. Ptasinska et al*.* pointed out that a transient phase PbI_2+*x*_^*x−*^ (0 ≤ x < 1) forms during the evolution of a hydrated complex under ambient conditions [96]. The resulting PbI_2+*x*_^*x−*^ is reactive when exposed to air and decomposes into lead-containing compounds inducing amorphous PbO, Pb(OH)_2_, and PbCO_3_ (Eqs. 2.1-9, 2.1-10, 2.1-11), which is evidenced by X-ray photoelectron spectra (XPS) characterizations in Fig. 2c.

2.1-6 $$\left({CH}_{3}{NH}_{3}\right){PbI}_{3}+{H}_{2}O\rightleftharpoons \left({CH}_{3}{NH}_{3}\right){PbI}_{3}\cdot {H}_{2}O$$

2.1-7 $$4\left({CH}_{3}{NH}_{3}\right){PbI}_{3}+{8H}_{2}O\to {{\left({CH}_{3}{NH}_{3}\right)}_{4}PbI}_{6}\cdot {2H}_{2}O+3Pb{\left(OH\right)}_{2}+6HI\uparrow $$

2.1-8 $${{\left({CH}_{3}{NH}_{3}\right)}_{4}PbI}_{6}\cdot {2H}_{2}O\to {{\left({CH}_{3}{NH}_{3}\right)}_{x}PbI}_{2+x}+\left(4-x\right){CH}_{3}{NH}_{2}\uparrow +\left(4-x\right)HI\uparrow +{2H}_{2}O$$

2.1-9 $$ 2\left( {CH_{3} NH_{3} } \right)_{x} PbI_{{2 + x}}  + 2CO_{2}  + O_{2}  \to 2PbCO_{3}  + 2I_{2}  + 2xCH_{3} NH_{2}  \uparrow  + 2xHI \uparrow   $$

2.1-10 $$ \left( {CH_{3} NH_{3} } \right)_{x} PbI_{{2 + x}}  + H_{2} O + \frac{1}{2}O_{2}  \to Pb\left( {OH} \right)_{2}  + I_{2}  + xCH_{3} NH_{2}  \uparrow  + xHI \uparrow   $$

2.1-11 $$Pb{\left(OH\right)}_{2}\to PbO+{H}_{2}O$$

**Fig. 3:**
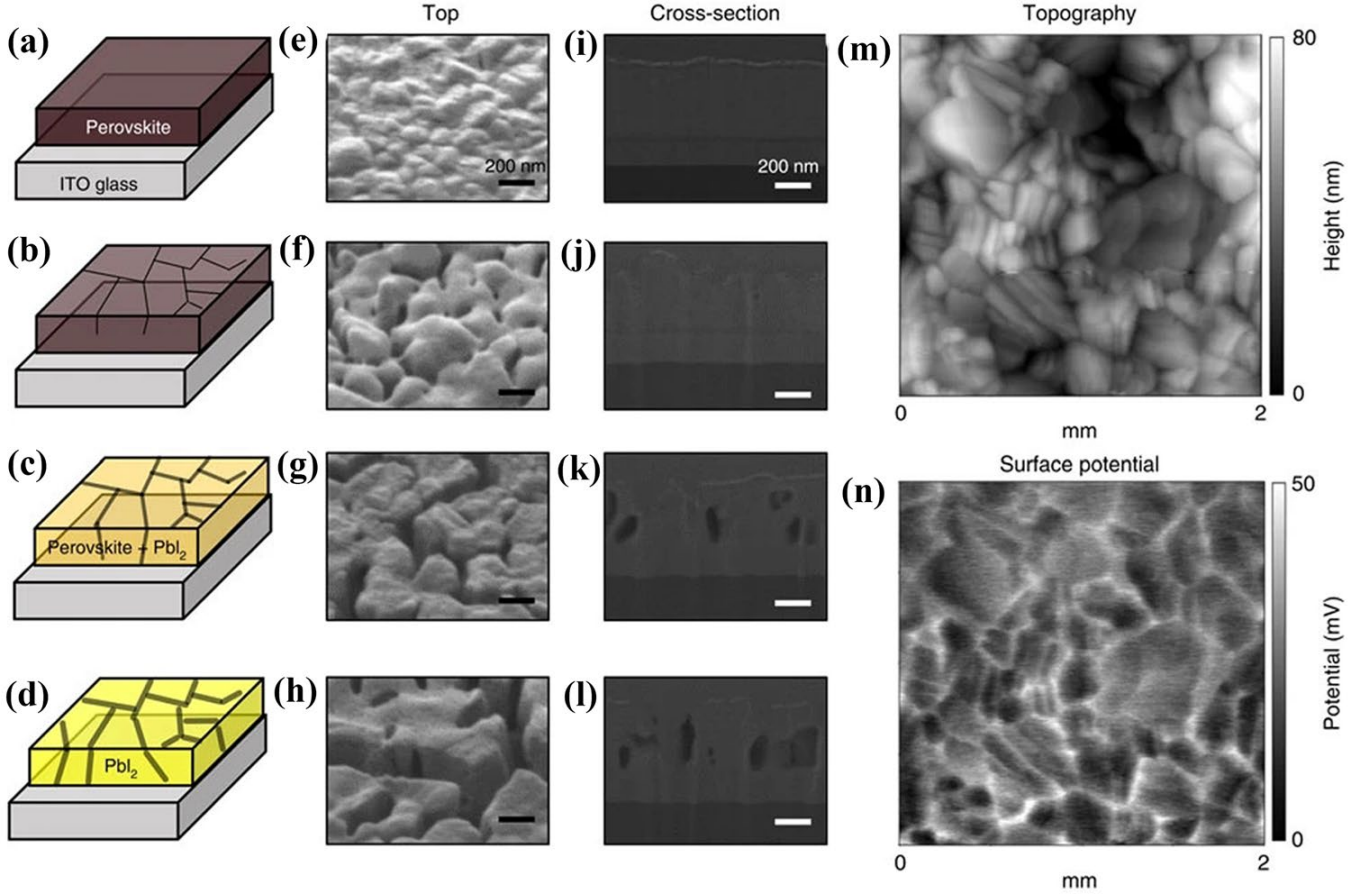
**a–d** Schematic illustration of perovskite degradation processes (left), **e–h** top-view (middle) and **i–l** cross-sectional (right) SEM images of perovskite layers (**a**,** e**,** i**) before, (**b**,** f**,** j**) after 6 h, (**c**,** g**,** k**) 12 h and (**d**,** h**,** l**) 18 h by ion deposition in humidified nitrogen. The color change from dark brown to yellow in **a-d** represents the gradual degradation process. Black lines and their widths in **a–d** represent grain boundaries and degradation extent, respectively. Scale bars, 200 nm. **m** Topography and **n** surface potential profile of MA_0.6_FA_0.4_PbI_2.9_Br_0.1_ film obtained from KPFM measurements after deposition of N_2_-positive ions. Reproduced with permission from Ref. [98]. Copyright 2016, Springer Nature. (Color figure online)

It’s reported that trapped charges can facilitate the degradation reactions of MAPbI_3_ in humid conditions. Ahn et al*.* deposited different polarity ions on the perovskite surfaces in humidified nitrogen and discovered that MAPbI_3_ is irreversibly decomposed to yellow PbI_2_ only when moisture and charges coexist (Fig. 3a–d) [97]. Figure 3e–l demonstrate that the degradation of perovskite starts from the GBs and the striking resemblance in kelvin probe force microscopy (KPFM) measurements (Fig. 3m and n) indicates that charges are preferentially trapped along GBs, which demonstrates that trapped charges can give rise to irreversible degradation. The degradation mechanism can be summarized: first, the perovskite material undergoes a hydration reaction in humid environment. Next, the organic cations, like MA^+^, will be deprotonated with the help of the local electric field induced by the charges trapped at the defect sites. The deprotonation process in the presence of water is shown below:

2.1-12 $$ CH_{3} NH_{3}^{ + }  + H_{2} O\xrightarrow{{TC}}CH_{3} NH_{2}  \uparrow  + H_{3} O^{ + }  $$

Here TC represents trapped charges. The release of gas phase CH_3_NH_2_ will shift the following hydration equilibrium reaction to the right side, causing the perovskite to start irreversible decomposition:

2.1-13 $$ \left( {CH_{3} NH_{3} } \right)PbI_{3}  + H_{2} O\xrightarrow{{TC}}PbI_{2}  + CH_{3} NH_{2}  \uparrow  + H_{3} O^{ + }  + I^{ - }  $$

In addition, oxygen can also accelerate the aging of perovskite in humid conditions, which is attributed to the scavenging action of O_2_ on the H_3_O^+^ proton formed in the aforementioned deprotonation process (Eq. 2.1-12). The overall degradation reaction of MAPbI_3_ with the participation of O_2_ is shown as follows:

2.1-14 $$ \left( {CH_{3} NH_{3} } \right)PbI_{3}  + \frac{1}{4}O_{2} \xrightarrow{{H_{2} O + TC}}PbI_{2}  + CH_{3} NH_{2}  \uparrow  + \frac{1}{2}H_{2} O + \frac{1}{2}I_{2}      $$

In summary, lead halide perovskite can easily decompose into PbI_2_, CH_3_NH_2_, HI, and other products in humid conditions with/without other factors (e.g. UV light, trapped charges, oxygen). The degradation reaction of MAPbI_3_ is usually irreversible due to the formed gas products (e.g. CH_3_NH_2_, HI) will release into the air. Therefore, protecting perovskites from water is crucial to improve the stability of PSCs.

#### Oxygen-Induced Reactions

**Fig. 4:**
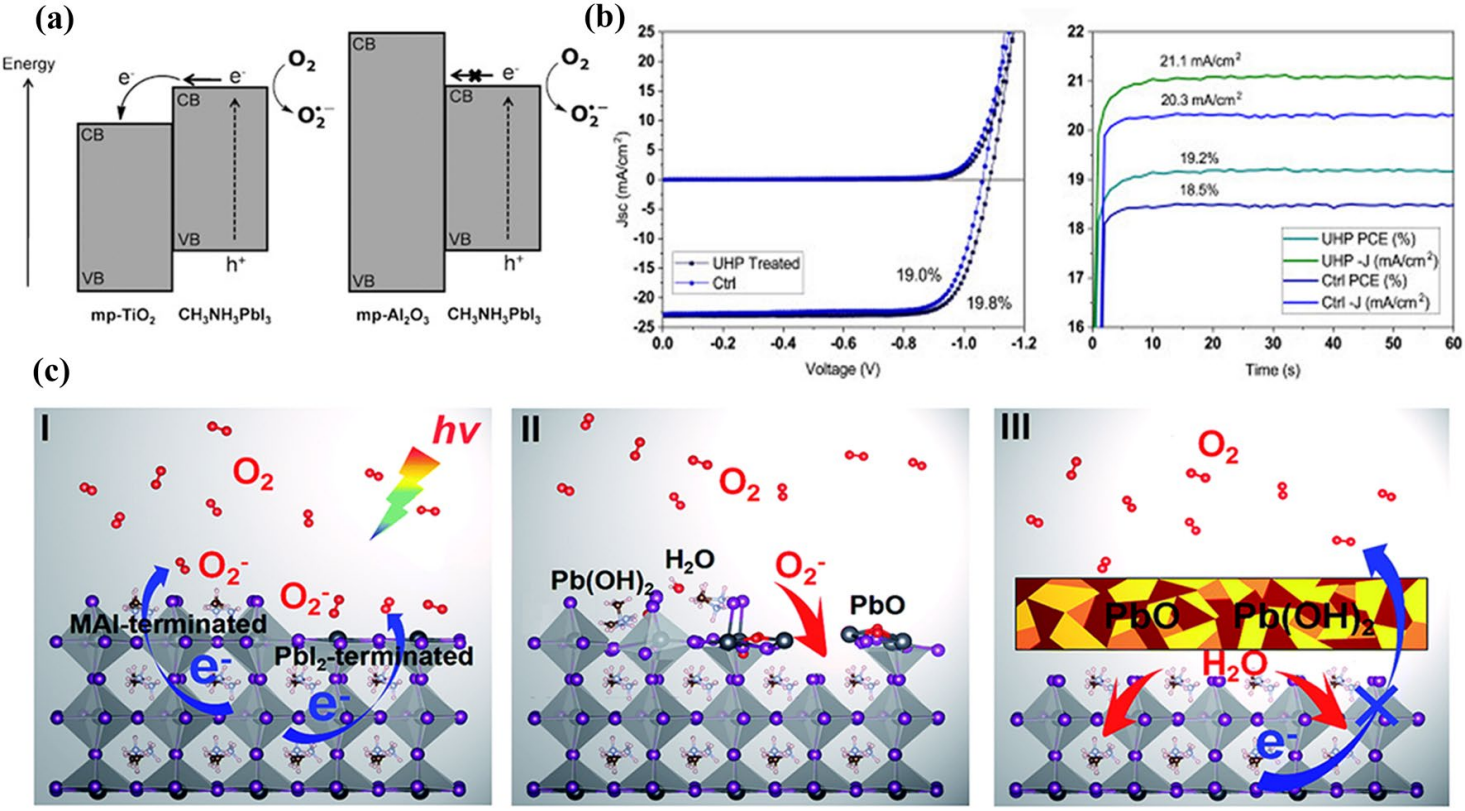
**a** Schematic model showing the electron transfer of the photoexcited electrons in the MAPbI_3_ layers to oxygen resulting in the formation of superoxide. Reproduced with permission from Ref. [101]. Copyright 2015, Wiley-VCH. **b** Photovoltaic performance characteristics of p-i-n PSC treated by H_2_O_2_ via the gas-phase deposition method with urea hydrogen peroxide for 40 s, compared to a control device, measured under AM1.5 100 mW/cm^2^ simulated sunlight. Reproduced with permission from Ref. [103]. Copyright 2019, Elsevier. **c** Schematic representation of the photo-oxidative degradation process of the MAPbI_3_ (001) surface. Reproduced with permission from Ref. [108]. Copyright 2019, Royal Society of Chemistry

Oxygen can induce the degradation of lead halide perovskites under certain conditions [98]. A study found that O_2_ molecules are only physically attached to the perovskites surfaces without chemical reactions. Once the adsorbed O_2_ captures an excess electron to form superoxide (O_2_^·−^), the degradation reactions of perovskites will proceed [99]. Haque et al*.* reported that O_2_ could permeate into the grain surface and interior of MAPbI_3_, which could also be reduced to highly reactive O_2_^·−^ with the help of photo-excited electrons [57, 100]. It’s observed that the Al_2_O_3_/MAPbI_3_ system can produce more O_2_^·−^ than TiO_2_/MAPbI_3_ system. In Fig. 4a, the TiO_2_ film can accept an electron from the photo-excited MAPbI_3_ because of the favorable energy offset at the heterojunction. Therefore, the fewer electrons transferring to oxygen leads to a lower yield of O_2_^·−^ for the TiO_2_/MAPbI_3_ system. Simultaneously, ab initio simulations demonstrate the O_2_^·−^ prefer energetically to occupy the iodide vacancies sites [101]. The photo-induced O_2_^·−^ formation is the key factor for the degradation reactions of MAPbI_3_:

2.1-15 $$ \left( {CH_{3} NH_{3} } \right)PbI_{3} \xrightarrow{{light}}\left( {CH_{3} NH_{3} } \right)PbI_{3}^{*}  $$

2.1-16 $$ O_{2} \xrightarrow{{\left( {CH_{3} NH_{3} } \right)PbI_{3}^{*} }}O_{2}^{{ \cdot  - }}  $$

2.1-17 $$ 4(CH_{3} NH_{3} )PbI_{3}^{*}  + O_{2}^{{( \cdot \, - )}}  \to 4PbI_{2}  + 2I_{2}  + 2H_{2} O + 4CH_{3} NH_{2}  \uparrow   $$

CH_3_NH_3_PbI_3_^*^ carries both photo-induced electrons and holes (Eq. 2.1-15), and O_2_ captures an electron from CH_3_NH_3_PbI_3_^*^ to form O_2_^·−^ (Eq. 2.1-16). The study found that MAPbI_3_ films with large grains degrade more slowly, which is attributed to large crystallites containing the fewer surface reaction sites. Based on the studies above, Sultana et al*.* discovered that the degradation product PbI_2_ (Eq. 2.1-17) could further react with O_2_ to produce lead oxyiodide under UV irradiation [38], which can be expressed as follows:

2.1-18 $$ PbI_{2} \xrightarrow{{UV}}e_{{cb}}^{ - } \left( {PbI_{2} } \right) + h_{{vb}}^{ + } \left( {PbI_{2} } \right) $$

2.1-19 $${e}_{cb}^{-}\left(Pb{I}_{2}\right)+{O}_{2}\to {O}_{2}^{\bullet -}+Pb{I}_{2}$$

2.1-20 $$\left(x+y\right)Pb{I}_{2}+\frac{y}{2}{O}_{2}^{\bullet -}\to {(Pb{I}_{2})}_{x}{(PbO)}_{y}+y{I}_{2}$$

The electron is excited from the valence band of PbI_2_ to its conduction band under UV irradiation (Eq. 2.1-18), which then transfers to O_2_ molecular to generate O_2_^·−^ (Eq. 2.1-19). Finally, the highly reactive O_2_^.−^ further reacts with PbI_2_ generating lead oxyiodide.

It’s reported that O_2_^.−^ will capture an acid proton of the CH_3_NH_3_^+^ to generate hydroperoxyl radical (HO_2_·) once approaching the ammonium group (Eq. 2.1-21) [102, 103]. Subsequently, two different reaction mechanisms are proposed to express next degradation process of perovskite. The first one is that two HO_2_· interact to produce hydrogen peroxide (H_2_O_2_) under lights (Eq. 2.1-22). Ultimately, H_2_O_2_ reacts with Pb^0^ on the perovskite surface generating PbO or Pb(OH)_2_ (Eqs. 2.1-23, 2.1-24). It is worth noting that the formed PbO has an unexpected passivation effect, preventing the accumulation of anion vacancies and formation of Pb-Pb dimers, which can increase the open-circuit voltage (*V*_oc_) for inverted PSCs as exhibited in Fig. 4b. The second one demonstrates that HO_2_· radicals can dissociate into H_2_ and O_2_, and the remaining O_2_ can serve as a reactant again for the continuous degradation process (Eq. 2.1-25).

2.1-21 $$ CH_{3} NH_{3}^{ + }  + O_{2}^{{ \bullet \, - }}  \leftrightarrow CH_{3} NH_{2}  \uparrow  + HO_{2}  \cdot     $$

2.1-22 $$ 2HO_{2}  \cdot  \leftrightarrow O_{2}  \uparrow  + H_{2} O_{2}  $$

2.1-23 $${P{b}^{0}+H}_{2}{O}_{2}\to Pb{(OH)}_{2}$$

2.1-24 $${P{b}^{0}+H}_{2}{O}_{2}\to PbO+{H}_{2}O$$

2.1-25 $$ 2HO_{2}  \cdot  \leftrightarrow 2O_{2}  \uparrow  + H_{2}  \uparrow   $$

Hillhouse et al*.* reported that H_2_O can accelerate the photooxidation reaction of MAPbI_3_ and proposed one plausible degradation pathway [104]. H_2_O rapidly undergoes the deprotonation reaction to form HO_2_· and HO^−^ in the presence of O_2_^·−^ (Eq. 2.1-26). As mentioned above, the newly formed HO_2_· will interact to release H_2_ and O_2_ (Eq. 2.1-25). Unfortunately, HO^−^ triggers the decomposition of MAPbI_3_ into PbI_2_ (Eq. 2.1-27), which subsequently reacts with H_2_O to generate PbIOH and HI (Eq. 2.1-28) in the following steps:

2.1-26 $$ O_{2}^{{( \bullet  - )}}  + H_{2} O \leftrightarrow HO^{ - }  + HO_{2}  \cdot   $$

2.1-27 $$\left({CH}_{3}{NH}_{3}\right){PbI}_{3}+H{O}^{-}\to {PbI}_{2}+{H}_{2}O+{CH}_{3}{NH}_{2}\uparrow +{I}^{-}$$

2.1-28 $$Pb{I}_{2}+{H}_{2}O\to PbIOH+HI\uparrow $$

It’s worth noting that O_2_^·−^ more rapidly reacts with H_2_O (Eq. 2.1-26) than CH_3_NH_3_^+^ (Eq. 2.1-21) due to a lower activation energy for the whole reaction pathway, which demonstrates that H_2_O can accelerate photooxidative degradation of MAPbI_3_.

Except for the degradation products mentioned above like PbI_2_ and lead oxyiodide, Snaith et al*.* detected metal lead (Pb^0^) on the perovskite surface [105] and proposed a plausible formation mechanism of Pb^0^ [102]. An iodide ion (I^−^) abstracts a photogenerated hole (h^+^) to produce an iodine atom (I^·^) (Eq. 2.1-29), which is accompanied by rapid site exchange of iodide from a regular to interstitial lattice site. Two iodine atoms combine with each other to generate I_2_, simultaneously leaving two iodine vacancies (V_I·_) (Eq. 2.1-30). Subsequently, V_I·_ captures an electron to generate Farbe center (V_I·_e’) (Eq. 2.1-31), which then reduces the Pb^2+^ in the adjacent site to Pb^+^ (Eq. 2.1-32). Finally, Pb^+^ undergoes a disproportionation reaction to produce Pb^0^ (Eq. 2.1-33).

2.1-29 $${I}^{-}+{h}^{+}\to {I}^{\bullet }$$

2.1-30 $$2{I}^{\bullet }\to {I}_{2}+2{V}_{{I}^{\bullet }}$$

2.1-31 $${V}_{{I}^{\bullet }}+{e}^{^{\prime}}\to {V}_{{I}^{\bullet }}{e}^{^{\prime}}$$

2.1-32 $${{Pb}^{2+}+V}_{{I}^{\bullet }}{e}^{^{\prime}}\to {V}_{{I}^{\bullet }}{Pb}^{+}$$

2.1-33 $$2{Pb}^{+}\to {Pb}^{2+}+{Pb}^{0}$$

Based on the above research, a photo-oxidative degradation mechanism of MAPbI_3_ is proposed for rapid surface oxidation and slow inner hydration [101, 106]. Wang et al*.* reported the three-step degradation process of MAPbI_3_ as displayed in Fig. 4c [107]. Step I: O_2_ on the MAPbI_3_ surface capture a photogenerated electron from the conduction band of MAPbI_3_ to form O_2_^−^ (Eq. 2.1-16). Step II: O_2_^−^ rapidly oxidizes the PbI_2_-terminated surface to produce PbO, resulting in a breakage of the Pb–I frameworks and the exposure of the MAI-terminated surface. Subsequently, the underlying MAI-terminated surface is further oxidized to generate H_2_O and PbO or the unstable Pb(OH)_2_. Step III: The oxidation products PbO and Pb(OH)_2_ can act as protective layers to inhibit the further oxidation of the internal MAPbI_3_. The newly produced H_2_O molecules from the surface oxidation and decomposition of Pb(OH)_2_ can slowly hydrate with the internal MAPbI_3_, finally leading to the structural collapse of MAPbI_3_.

In conclusion, the oxygen-induced reactions of perovskites need the participation of light or water. Firstly, O_2_ captures photogenerated electrons generating O_2_^−^, which subsequently introduces a series of decomposition pathways under different conditions. Finally, the generating degradation products mainly include CH_3_NH_2_, I_2_, PbI_2_, and PbO (or Pb(OH)_2_).

#### Light-Induced Reactions

**Fig. 5:**
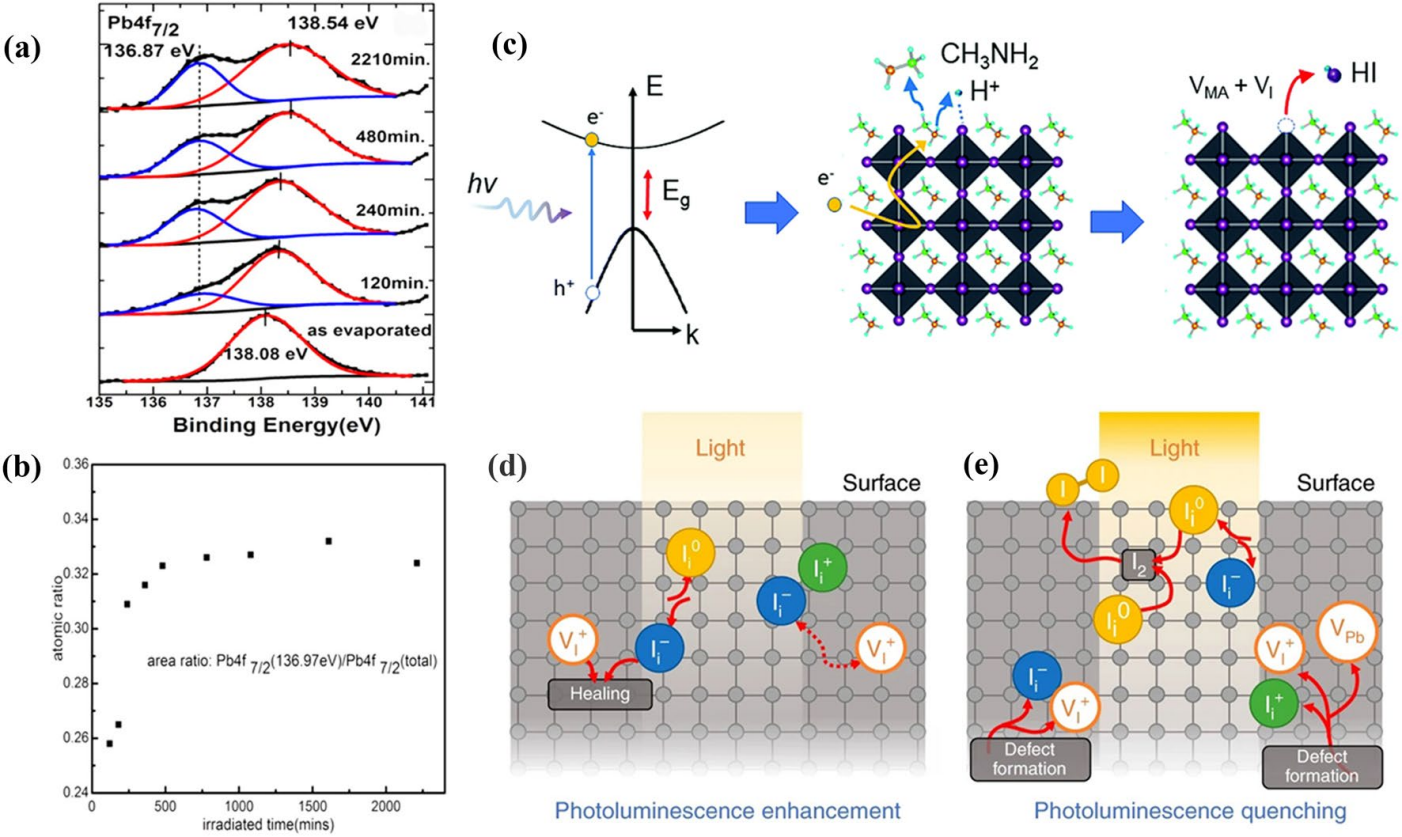
** a** Pb 4f7/2 decomposition and **b** metallic Pb fraction during laser irradiation. **a**,** b** Reproduced with permission from Ref. [111]. Copyright 2017, American Chemical Society. **c** Schematic representation of photodegradation mechanism. 1) Hot carrier generation, 2) deprotonation and release of MA gas, and 3) formation of HI gas and V_I_. Reproduced with permission from Ref. [112]. Copyright 2018, Royal Society of Chemistry. **d, e**, Photoluminescence enhancement and quenching mechanisms. Ion dynamics in MAPbI_3_ thin film promoting PLIE in **d**, when the probability of I_0_ species encounters is small and Frenkel pair annihilation is boosted by electron trapping, and PLID in **e**, when the probability of I_0_ species encounters is high, boosting I_2_ molecule formation. **d**, **e** Reproduced with permission from Ref. [113]. Copyright 2019, Springer Nature

Unlike oxygen, light can induce the perovskites degradation without any presence of other environmental factors (e.g., water, oxygen). Study demonstrated that UV light can induce severe degradation of lead halide perovskites [108, 109]. As shown in Fig. 5a, Gao et al*.* detected Pb^0^ on the MAPbI_3_ surface after about 120 min of UV irradiation with a wavelength of 408 nm [110]. The proposed degradation reactions of MAPbI_3_ under UV irradiation can be generalized in Eqs. 2.1-34, 2.1-35. Figure 5b shows that the ratio of Pb^0^ remains nearly constant after 480 min of UV irradiation, which indicates that the decomposition of MAPbI_3_ has already saturated.

2.1-34 $$ \left( {CH_{3} NH_{3} } \right)PbI_{3} \xrightarrow{{UV}}PbI_{2}  + HI \uparrow  + CH_{3} NH_{2}  \uparrow   $$

2.1-35 $$ PbI_{2} \xrightarrow{{UV}}Pb^{0}  + I_{2}  $$

Yan et al*.* proposed a possible photodegradation mechanism of MAPbI_3_ concerning hot carriers [111]. As shown in Fig. 5c, the first step is that MAPbI_3_ is excited by light (*hv* > 3 eV) to generate long-lived hot carriers (Eq. 2.1-36). Then a hot electron reacts with the CH_3_NH_3_^+^ via columbic coupling to generate a free proton and release CH_3_NH_2_ near surface or GB regions (Eq. 2.1-37). Finally, the free protons interact with undercoordinated I^−^ on the surface, generating volatile HI and iodine vacancies (Eq. 2.1-38). The release of HI can induce the destruction of Pb-I framework from the corner-shared to the face-shared Pb-I octahedral, causing the degradation of MAPbI_3_ into PbI_2_.

2.1-36 $$hv\left(>3\mathrm{eV}\right)\to {e}^{-}\left(hot\right)+{h}^{+}(hot)$$

2.1-37 $$ CH_{3} NH_{3}^{ + }  + e^{ - } (hot) \to CH_{3} NH_{2}  + H^{ + }  + e^{ - }  $$

2.1-38 $${I}^{-}+{H}^{+}\to HI\uparrow +{V}_{I}$$

It’s reported that halide defects have an influence on the photostability of MAPbI_3_ [112]. Petrozza et al*.* found that long-living carrier traps associated with halide defects could trigger photoconversion, which drives both photoluminescence intensity enhancement (PLIE) and photoluminescence intensity decrease (PLID). In Fig. 5d and e, PLIE and PLID processes are proposed to explain the observations. The timescales involved in PLID and PLIE are consistent with the reported ionic activities, such as ion/defect annihilation and migration rates [113, 114]. Generally, vacancies (e.g., *V*_Pb_) and interstitial halogen (e.g., I_i_) defects dominate in the perovskites. *V*_Pb_ is only moderately active as a trap, while I_i_ can remarkably trap both electrons and holes via (+ /0) and (0/ −) transitions. When the probability of encountering I^0^ species is very low, the light-induced PLIE is associated with annihilation of the I_i_^−^V_I_^+^ Frenkel pair:

2.1-39 $$ I_{i}^{ + } /I_{i}^{ - } ....V_{I}^{ + }  + e^{ - } \xrightarrow{{light}}I_{i}^{0} /I_{i}^{ - } ....V_{I}^{ + } \xrightarrow{{light}}I_{i}^{0}  + pristine\;material $$

The PLID mechanism is proposed to be a bimolecular reaction boosted by increasing the encountering probability of I^0^ species, occurring near the film surface filled with long-lived traps:

2.1-40 $$2{I}_{i}^{0}\stackrel{light}{\to }{I}_{2}$$

The two processes may coexist and compete in the perovskite material. If traps densities are adequately low, photo-induced PLIE will be a dominant effect. However, photo-induced PLID will play a key role when high-density traps appear near the film surface that may act as a reservoir for photogenerated species, eventually causing perovskites degradation. Therefore, passivating under-coordinated surface sites can prohibit defect formation and hence enhance the photostability of perovskites.

### Charge Transport Layers

In addition to the aforementioned degradation reactions induced by environmental factors, the chemical stability of lead halide PSCs can be affected by charge transport layers, including ETL and HTL. Generally, the interfacial chemical reactions are not desirable, which could lead to destroyed structure and reduced charge transport/extraction. However, for certain charge transport layers such as Cl-containing SnO_2_, interfacial reactions have been demonstrated to be beneficial for charge transport and chemical stability of PSCs. Considering the diversity of charge transport materials, a variety of interfacial reactions and their effects on device stability have been reported in literature. Therefore, the chemical reactions in PSCs induced by charge transport layers are elucidated in this session.

#### ETL-Induced Reactions

**Fig. 6:**
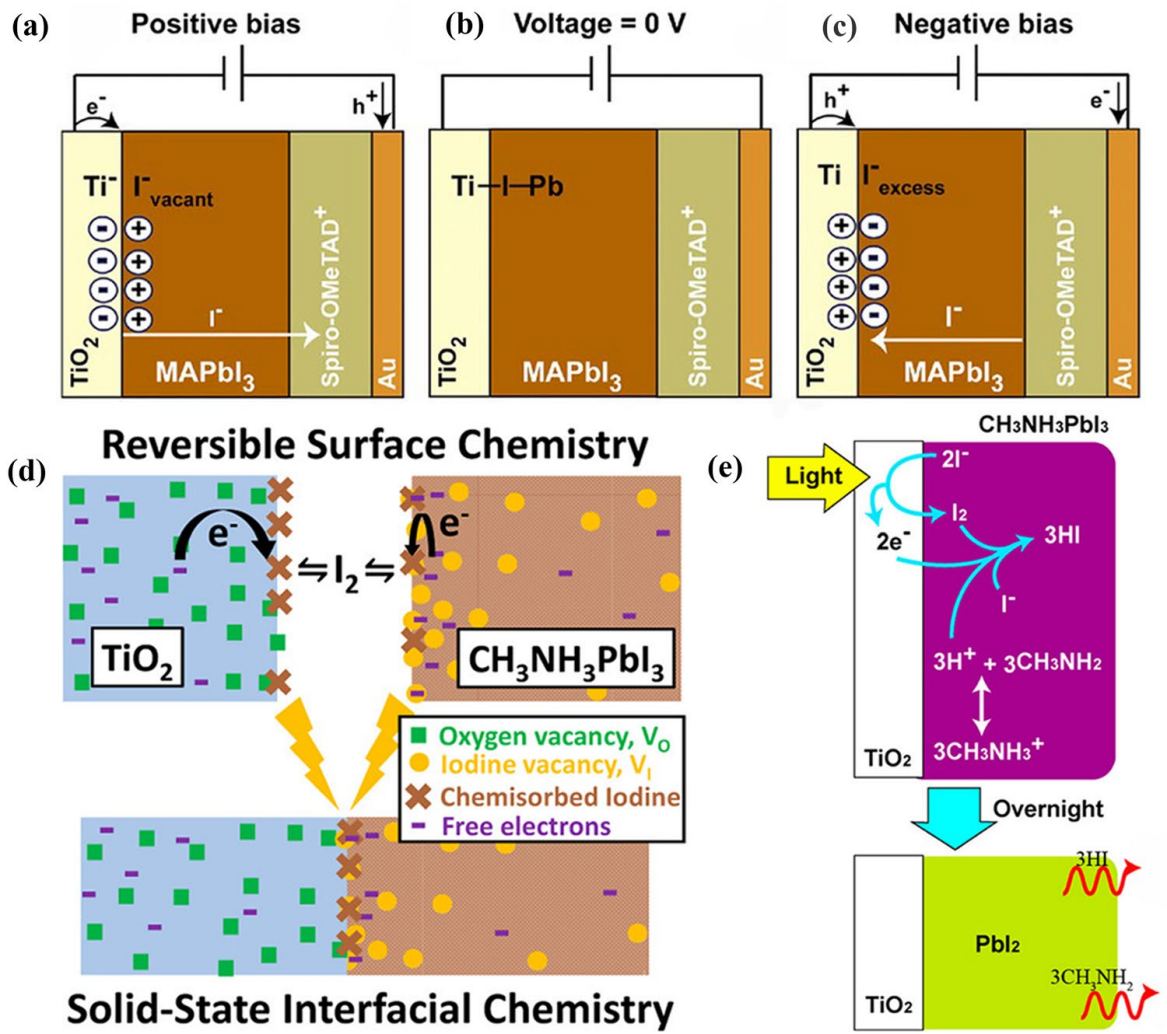
Diagram representing iodide migration and chemical species present at the interfaces. **a **At positive-bias iodine ions are forced to migrate toward the hole selective contact where the reaction with spiro-OMeTAD^+^ occurs. The iodide defective layer is formed at the TiO_2_/MAPbI_3_ interface. **b** At zero-bias the neutral case appears. **c** At negative-bias spiro-OMeTAD only partially returns to its oxidized, conductive state. Iodide ions accumulate at the TiO_2_/perovskite interface. **a-c** Reproduced with permission from Ref. [49]. Copyright 2016, Wiley-VCH. **d** Scheme showing the reversible chemistry reactions process occurring at the solid-state TiO_2_/CH_3_NH_3_PbI_3_ interface. Reproduced with permission from Ref. [124]. Copyright 2017, American Chemical Society. **e** Degradation scheme of CH_3_NH_3_PbI_3_ perovskite solar cells during light exposure test: TiO_2_/CH_3_NH_3_PbI_3_. Reproduced with permission from Ref. [125]. Copyright 2014, American Chemical Society

TiO_2_ has been widely used as an electron transport material in the early study of PSCs [36, 115–121]. Belmonte et al*.* reported that TiO_2_ interacts with MAPbI_3_ mainly through binding I^−^ in MAPbI_3_ to undercoordinated Ti^4+^ in TiO_2_ [49, 122]. Notably, this Ti-I-Pb bond is not strong due to the little hybridization between Pb_*s–p*_ state and Ti_*d*_ orbital, hence the adsorption/desorption of I^−^ at the interface may occur easily. The interactions between TiO_2_ and perovskite are reversible under both positive and negative biases, as shown in Fig. 6a–c. At a positive bias, weakly bonded I^−^ migrate towards the hole transport layer contact and hence I vacancies remain at MAPbI_3_/TiO_2_ interface, and the positive charges are compensated by electrons injection and accumulations at TiO_2_. In contrast, excess I^−^ may accumulate at MAPbI_3_/TiO_2_ interface under a negative bias. The Ti-I-Pb bonds easily accommodate excessive defect or ion charges in a highly reversible manner to generate capacitive currents. However, it's worth noting that this reversible interaction has a negligible effect on the photovoltaic performance and chemical stability of PSCs.

Studies on film’s current response to I_2_ vapor have revealed that reversible chemical reactions occur at TiO2/I_2_ and MAPbI3/I_2_ interfaces [123]. Rand et al*.* discovered the pathways for I_2_ passivation of MAPbI_3_ surface defects from the photoconductivity data of MAPbI_3_ films. According to the current response of TiO_2_ film to I_2_ vapor pressures, they proposed a plausible reaction mechanism between TiO_2_ and I_2_. As shown in Eqs. 2.2-1, 2.2-2, and 2.2-3, TiO_2_ and MAPbI_3_ surfaces undergo strong reversible reactions with I_2_ gas, respectively. Combining these three reactions, the chemical interaction between TiO_2_ and MAPbI_3_ can be generalized in Eq. 2.2-4.

2.2-1 $${e}_{MAPb{I}_{3}}^{-}+{trap}_{MAPb{I}_{3}}^{0}\rightleftharpoons {trap}^{-}$$

2.2-2 $${trap}^{-}+\frac{1}{2}{I}_{2}\rightleftharpoons {I}_{adsorbed(MAPb{I}_{3})}^{-}$$

2.2-3 $${e}_{Ti{O}_{2}}^{-}+\frac{1}{2}{I}_{2}\rightleftharpoons {I}_{adsorbed(Ti{O}_{2})}^{-}$$

2.2-4 $${e}_{Ti{O}_{2}}^{-}+{I}_{adsorbed(MAPb{I}_{3})}^{-}\rightleftharpoons {I}_{adsorbed\left(Ti{O}_{2}\right)}^{-}+{e}_{MAPb{I}_{3}}^{-}+{trap}_{MAPb{I}_{3}}^{0}$$

I^−^ in MAPbI_3_ is adsorbed on the TiO_2_ surface and deplete an electron on TiO_2_, leaving a free electron as well as a trap on the MAPbI_3_ surface (Fig. 6d). The energy level of the trap would determine whether the electron is captured or freely transmitted. Therefore, MAPbI_3_ may be chemically reduced by TiO_2_ due to the differences in work function. In addition, the newly formed traps on the MAPbI_3_ surface will make a difference to carrier density and recombination rate, which could deteriorate the device stability.

TiO_2_ was also reported to catalyze MAPbI_3_ decomposition in the presence of light [124]. In Fig. 6e, TiO_2_ extracts an electron from I^−^ under light irradiation generating I_2_ (Eq. 2.2-5), which deconstructs perovskites crystal and reduces their chemical stability. As mentioned in *2.2.1* part, CH_3_NH_3_^+^ can be deprotonated to generate CH_3_NH_2_ and H^+^ in humid conditions. The presence of I_2_ and H^+^ will accelerate the reaction (Eq. 2.2-6) to proceed forward, further destroying the perovskite structure.

2.2-5 $$ 2I^{ - } \xrightarrow{{TiO_{2}  + light}}I_{2}  + 2e_{{TiO_{2} }}^{ - }  $$

2.2-6 $${{I}^{-}+I}_{2}+3{H}^{+}+2{e}_{Ti{O}_{2}}^{-}\rightleftharpoons 3HI\uparrow $$

TiO_2_ acts as a catalyzer rather than a reagent in the reactions with perovskites, which accelerates the degradation process of perovskites to some extent. Therefore, reducing the catalytic performance of TiO_2_ is an effective way to improve the photovoltaic performance and chemical stability of TiO_2_-based PSCs.

**Fig. 7:**
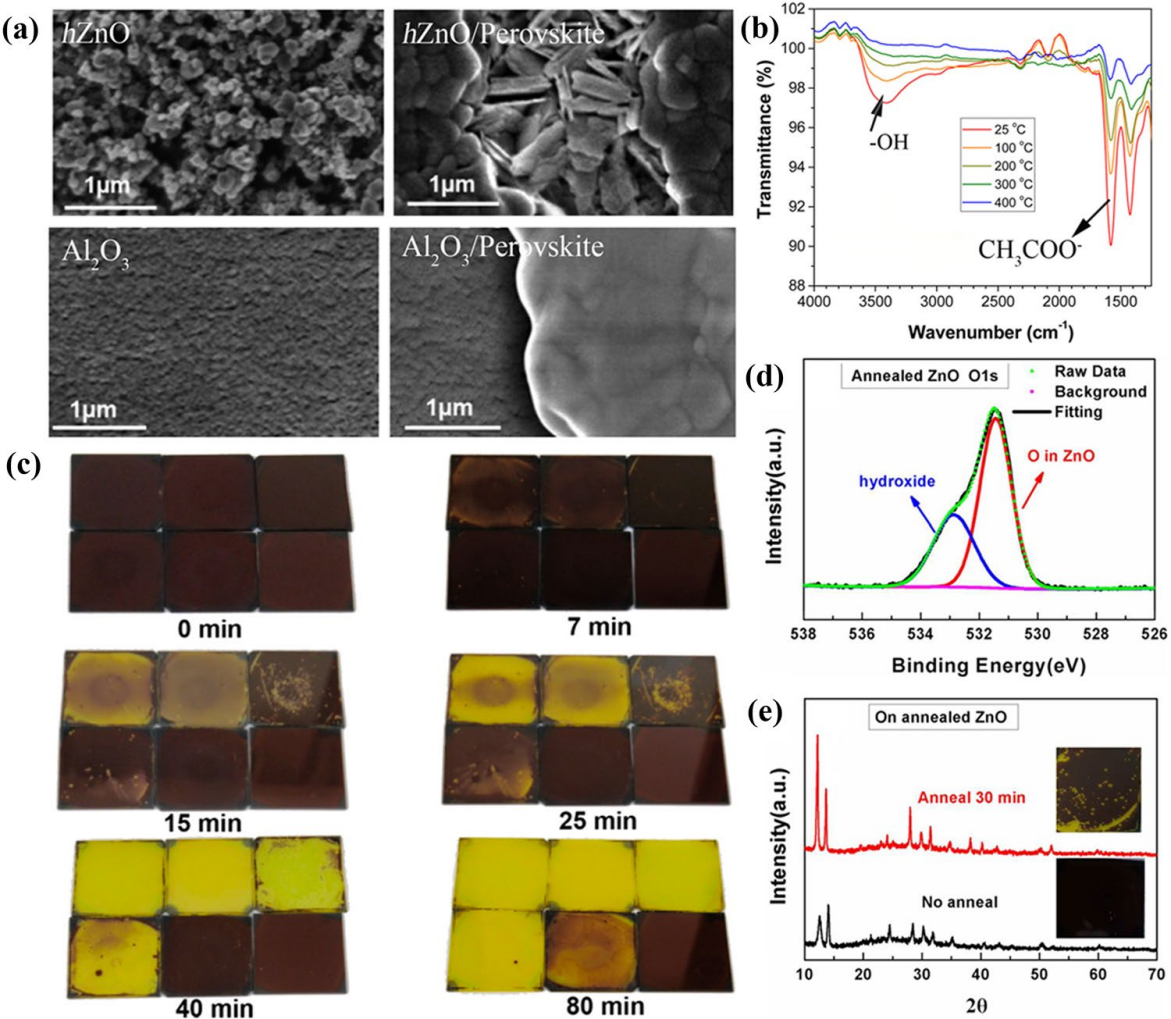
** a** SEM of *h*ZnO and Al_2_O_3_ substrates before (left) and after (right) deposition of perovskite layer. Reproduced with permission from Ref. [131]. Copyright 2016, Royal Society of Chemistry. **b** FTIR spectra of ZnO films on glass annealed at various temperatures. **c **Photographs of CH_3_NH_3_PbI_3_ films deposited on thermally pretreated ZnO layers and heated to 100 °C for the indicated time: top row (left to right): pretreated at 25, 100, 200 °C. Bottom row (left to right): pretreated at 300 °C, 400 °C, and SiO_2_/CH_3_NH_3_PbI_3_. **b**, **c** Reproduced with permission from Ref. [132]. Copyright 2015, American Chemical Society. **d** High-resolution of O 1*s *XPS core level spectra of ZnO with annealing at 200 °C for 1 h in air. **e** XRD patterns and photographs of perovskite film on annealed ZnO without thermal annealing and annealing at 100 °C for 30 min. **d**, **e** Reproduced with permission from Ref. [133]. Copyright 2015, American Chemical Society. (Color figure online)

It’s reported that ZnO ETL can also interact chemically with perovskites [125–129]. Anta et al*.* proposed that an acid–base reaction could occur at the ZnO/perovskite interface [130], triggering the decomposition of perovskite into PbI_2_ in humid conditions. In addition, this interaction could cause redissolution of the ZnO substrate, whose morphology changes from spherical nanoparticles to aciculate particles (Fig. 7a).

Additionally, the deprotonation reaction between ZnO and perovskite can cause the thermal degradation of perovskite. Kelly et al*.* found that the basic hydroxyl groups and residual acetate ligands exist on the ZnO surface through the Fourier transform infrared spectroscopy (FTIR) spectra analysis (Fig. 7b), which can be eliminated or reduced by high-temperature calcination of ZnO films (Fig. 7c) [131]. Otherwise, the hydroxide or residual acetate ligands will react with CH_3_NH_3_^+^ destroying the crystal structure of perovskite, which is evidenced in Fig. 7d and e [132]. And this decomposition process of MAPbI_3_ can be expressed as follows:

2.2-7 $${OH}^{-}{+CH}_{3}{NH}_{3}^{+}\to {CH}_{3}{NH}_{3}OH$$

2.2-8 $$ CH_{3} NH_{3} OH\xrightarrow{{heat}}CH_{3} NH_{2}  \uparrow  + H_{2} O $$

As shown in Eqs. 2.2-7 and 2.2-8, the whole degradation reactions are the deprotonation process of CH_3_NH_3_^+^, and CH_3_NH_3_OH easily decomposes into CH_3_NH_2_ gas and H_2_O under heat. The decomposition of CH_3_NH_3_OH can promote the reaction (Eq. 2.2-7) to proceed forward continuously, which accelerates MAPbI_3_ degradation. It’s reported that MAPbI_3_ decomposes easily to form HI in humid conditions [94], which subsequently reacts with ZnO resulting in reduced electron mobility of ZnO and deteriorative chemical stability of PSCs. This neutralization reaction can be presented in the following reaction [127]:

49 $$2HI+ZnO\to Zn{I}_{2}+{H}_{2}O$$

**Fig. 8:**
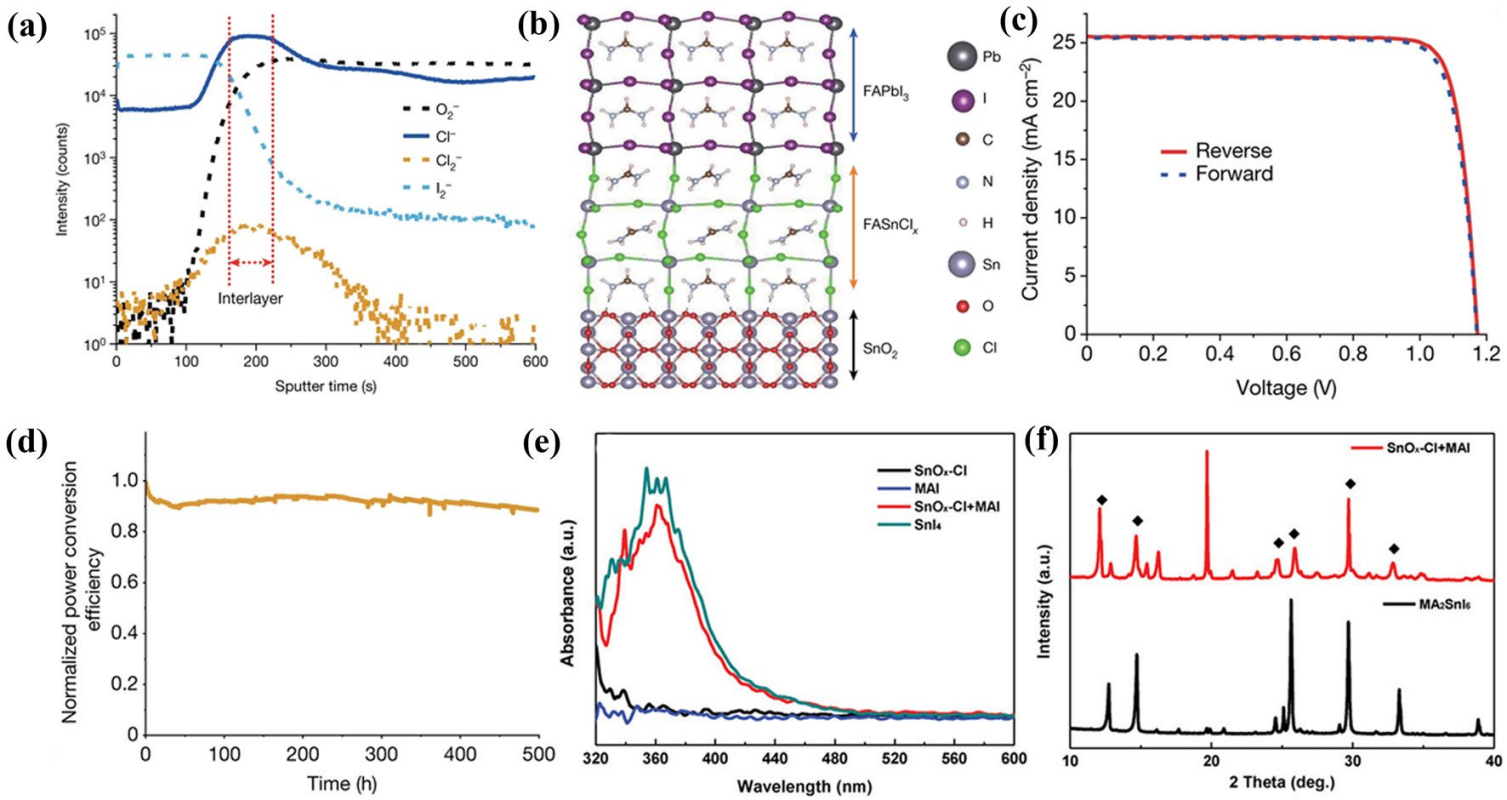
**a** ToF-SIMS depth profiles for the perovskite and Cl-bonded SnO_2_ on FTO. **b** Simulation of the formation of the FASnCl_x_ interlayer between perovskite and SnO_2_. **c** *J*-*V* curves of the best-performing device, measured in reverse (red solid line) and forward (blue dashed line) modes. **d** Maximum power point tracking measured for the PSC fabricated using Cl-bonded SnO_2_ and Cl-containing FAPbI_3_. **a-d** Reproduced with permission from Ref. [5]. Copyright 2021, Springer Nature. **e** UV-vis spectra of SnO_x_-Cl, MAI, SnO_x_-Cl+MAI, and SnI_4_, respectively. **f** XRD pattern of SnO_x_-Cl + MAI and reference MA_2_SnI_6_ powder samples, providing the feasibility of the ion-exchange reaction. **e**, **f** Reproduced with permission from Ref. [136]. Copyright 2019, Wiley-VCH. (Color figure online)

SnO_2_ is widely used in PSCs owing to its good electron transport ability and chemical stability. However, the interactions between the Cl-containing SnO_2_ and perovskite still exist. Recently, Seok et al*.* reported that a FASnCl_x_ interlayer at a SnO_2_/perovskite interface could be formed by an interfacial reaction between Cl-bonded SnO_2_ and Cl-containing FAPbI_3_ perovskite (Fig. 8a and b) [5], which is related to the easy formation of Sn-based perovskites in the presence of Cl^−^, FA^+^ and Sn^2+^ [133, 134]. The coherent interlayer reduces the interfacial charge recombination and enhances charge transport/extraction, achieving stable PSCs with a high PCE of 25.8% (Fig. 8c and d). However, Sn-Cl bonds did not form when the Cl-containing FAPbI_3_ solution was applied on a pure SnO_2_ surface. In comparison, Cl-bonded SnO_2_ can interact with Cl^−^-free FAPbI_3_ precursor to form Sn-I bonds by Cl^−^–I^−^ exchange. In addition, Pang et al*.* also discovered the spontaneous ion-exchange reaction between Cl^−^ and I^−^ at the SnO_x_-Cl/MAPbI_3_ interface (Fig. 8e and f), which could effectively passivate the physical contact defects. The diffusion of Cl^−^ in the MAPbI_3_ films promoted the grain longitudinal growth and decreased the GB density [135]. It is worth noting that the reactions between SnO_x_–Cl and perovskite effectively passivate the interface defects, thereby improving the photovoltaic performance and chemical stability of the PSCs.

Fullerene derivatives, like phenyl-C_61_-butyric acid methyl ester (PCBM) and [6, 6]-Phenyl-C71-butyric acid methyl ester (PC_71_BM), are another type of popularly used electron transport materials particularly in inverted PSCs. It’s reported that PCBM undergoes an electron transfer reaction with halogens in perovskites to produce PCBM-nX radicals [74, 136]. Additionally, halogens favorably interact with C_70_ face rather than O face of PC_71_BM through covalent bonds [137]. Such strong interactions can suppress Pb-I antisite defects and ions migration at perovskite/PCBM interfaces, which can lead to improved stability and reduced hysteresis of PSCs [138]. However, C_60_, another commonly used ETL in the inverted PSCs, barely undergoes interfacial chemical reactions with perovskites because of its chemical inertness.

#### HTL-Induced Reactions

**Fig. 9:**
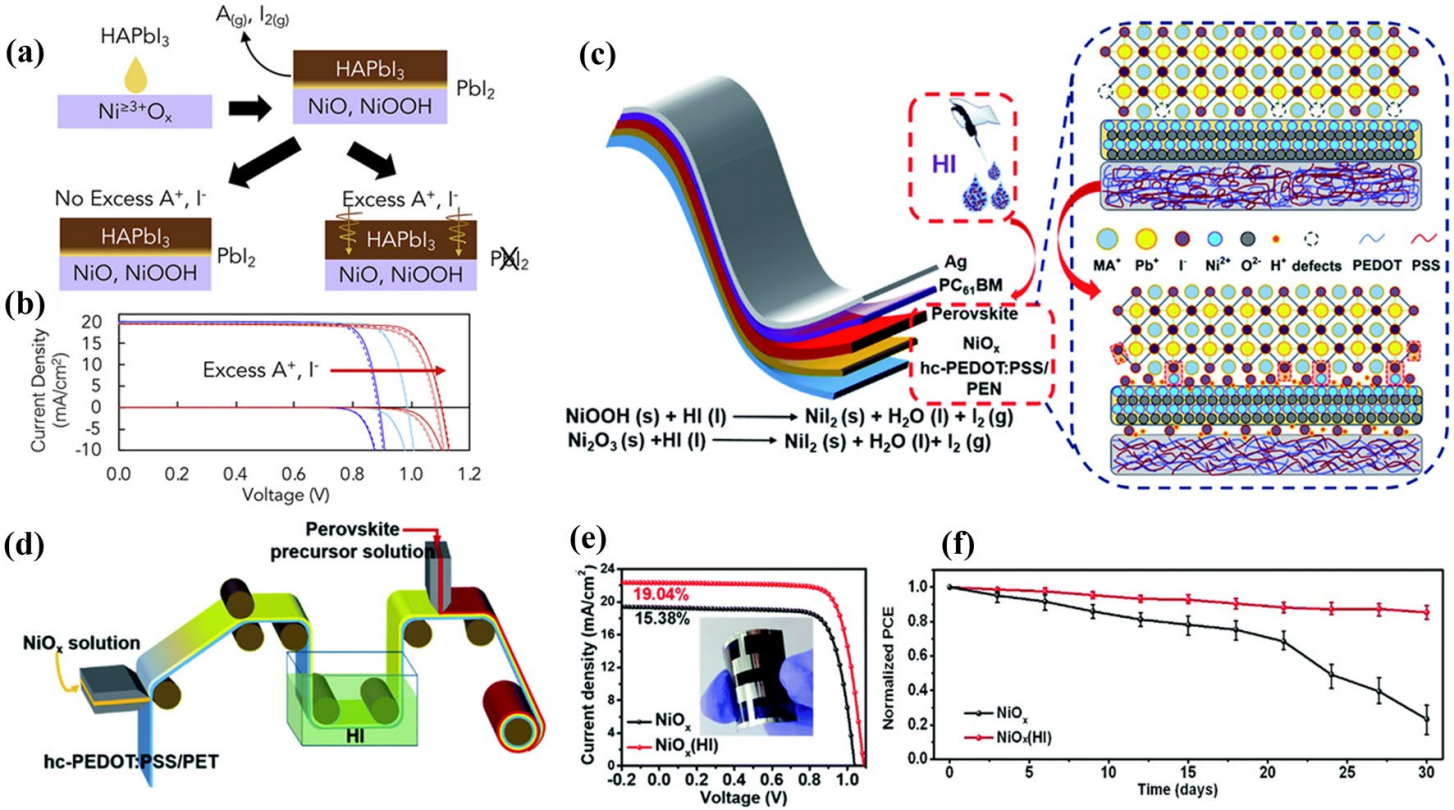
**a** Schematic of the formation process of PbI_2-x_Br_x_ at the interface. **b** Dark and light *J*-*V* curves of Cs_0.25_FA_0.75_Pb(Br_0.2_I_0.8_)_3_ perovskite solar cells with 0–3 mol% excess A-site. **a**,** b** Reproduced with permission from Ref. [150]. Copyright 2020, Elsevier. **c** Diagram of the passivation mechanism. **d** Fabrication procedure for perovskite solar cells by R2R process. **e** *J*-*V* curves of flexible devices (1.01 cm^2^) with and without HI treatment. Inset presents the photograph of flexible perovskite solar cells. **f** Normalized PCE of encapsulated PSCs based on NiO_x_ and NiO_x_ (HI) hole-transport layers under ambient condition (25–55% relative humidity and 25–35 ℃) for 30 days. **c-f** Reproduced with permission from Ref. [146]. Copyright 2021, Royal Society of Chemistry

In general, the widely used HTLs in inverted PSCs (i.e., p-i-n structure) are mainly poly(3,4-ethylenedioxythiophene) polystyrene sulfonate (PEDOT:PSS) [139, 140], poly[bis(4-phenyl)(2,4,6-trimethylphenyl)amine (PTAA) [141–143] and NiO_x_ [144, 145]. As an inorganic HTL, NiO_x_ is regarded as a promising HTL candidate with the advantages of good optical transparency, excellent stability and low cost [146, 147]. The interaction between NiO_x_ and perovskite has been studied by many groups. McGehee et al*.* proposed a surface-assisted electron transfer-proton transfer (ET-PT) reaction mechanism at the NiO_x_/perovskite interface [148]. They deposited various perovskite precursors (MAI, MABr, MACl, CsI, and PbI_2_ in acetonitrile solvent, respectively) onto NiO_x_ surface and observed that only MAI could bleach NiO_x_ film, which demonstrates that Ni^≥3+^ sites can oxidize I^−^ only in the presence of a proton donor but neither Br^−^ or Cl^−^ because of their much higher oxidation potentials. In conclusion, Ni^≥3+^ defect sites can act not only as Lewis acid to oxidize I^−^ but also as Brønsted base to deprotonate CH_3_NH_3_^+^. The perovskite solution was deposited on the various HTLs (PTAA, poly-TPD, and NiO_x_) to detect the differences in the perovskite films through XRD analysis. It’s observed that PbI_2_ accumulates at the NiO_x_/perovskite interface or scatters throughout PTAA-based perovskite. However, only the interfacial PbI_2_ can block the extraction of holes resulting in the *V*_*oc*_ loss of PSCs. The whole reaction can be shown in Fig. 9a.

2.2-10 $$(C{H}_{3}{NH}_{3})Pb{I}_{3}+{Ni}^{\ge 3+}{O}_{x}\leftrightarrow {Ni}^{\ge 2+}{O}_{x}H+{PbI}_{2}+{CH}_{3}{NH}_{2}\uparrow +\frac{1}{2}{I}_{2}$$

According to reaction Eq. 2.2-10, the resulting A-site deficient in this region increases interface recombination and reduces chemical stability of PSCs. The study found that 1–5 mol% excess A-site cations were incorporated into the perovskite precursor solution to prevent the reaction above. Therefore, the formation of PbI_2_ layer at the NiO_x_/perovskite interface is inhabited, and the *V*_oc_ was improved by > 200 mV as shown in Fig. 9b.

Wang et al. reported that trivalent nickel compound (NiOOH) on NiO_x_ HTL surfaces can be reduced to nickel iodide (NiI_2_) by soaking the NiO_x_ HTLs in hydroiodic acid (HI) during roll-to-roll printing of flexible PSCs [144], as shown in Fig. 9c and d, which can enhance NiO_x_/perovskite interface contact and ameliorate the work function of NiO_x_ film. The reaction process is shown below:

2.2-11 $$NiOOH+3HI\to Ni{I}_{2}+2{H}_{2}O+{\frac{1}{2}I}_{2}$$

2.2-12 $$N{i}_{2}{O}_{3}+6HI\to 2Ni{I}_{2}+3{H}_{2}O+{I}_{2}$$

Subsequently, I^−^ in NiI_2_ can coordinate with Pb in perovskite to form a Pb-I bond, inducing an orderly growth of perovskite lattice and enhancing the crystallinity of the perovskite film. Consequently, flexible PSCs with improved PCE as well as remarkable chemical stability were achieved (Fig. 9e and f).

In addition to NiO_x_, the commonly used HTLs are PEDOT:PSS and PTAA in inverted PSCs. We note that PEDOT:PSS and PTAA are rarely reported to react with perovskites. However, PEDOT:PSS has been shown to corrode ITO due to its acidic composition, which can react with In_2_O_3_ [13, 139, 149, 150]. As a consequence, the dissociated indium ions can diffuse into the perovskite layer and then deteriorate device performance and stability [151, 152].

The normal PSCs (i.e. n-i-p structure) using 2,2',7,7'-Tetrakis[N,N-di(4-methoxyphenyl)amino]-9,9'-spirobifluorene (spiro-OMeTAD) as HTL have achieved efficiency records, however, the instability of spiro-OMeTAD itself and its additives limits their commercial applications [153]. Therefore, the interfacial reactions between perovskite and spiro-OMeTAD under various conditions have been investigated to improve the device stability. Sultana et al*.* detected the signal of [spiro-OMeTAD(PbI_2_)_n_PbI]^+^ adducts in the mass spectra and found that the interaction between spiro-OMeTAD and perovskite was beneficial for PSCs operations [38]. In addition, Belmonte et al*.* reported that I^−^ is driven towards the spiro-OMeTAD HTL and react with the oxidized spiro-OMeTAD^+^ under a positive bias (Eq. 2.2-13) [49]. This irreversible reaction turns the spiro-OMeTAD^+^ into neutral molecule, which prevents the oxidation of spiro-OMeTAD [154] and consequently decreases the conductivity of HTL. Hence, this process has a negative effect on the photovoltaic performance and chemical stability of PSCs.

2.2-13 $$spiro-OMeTA{D}^{+}+{I}^{-}\to spiro-OMeTAD-I$$

**Fig. 10:**
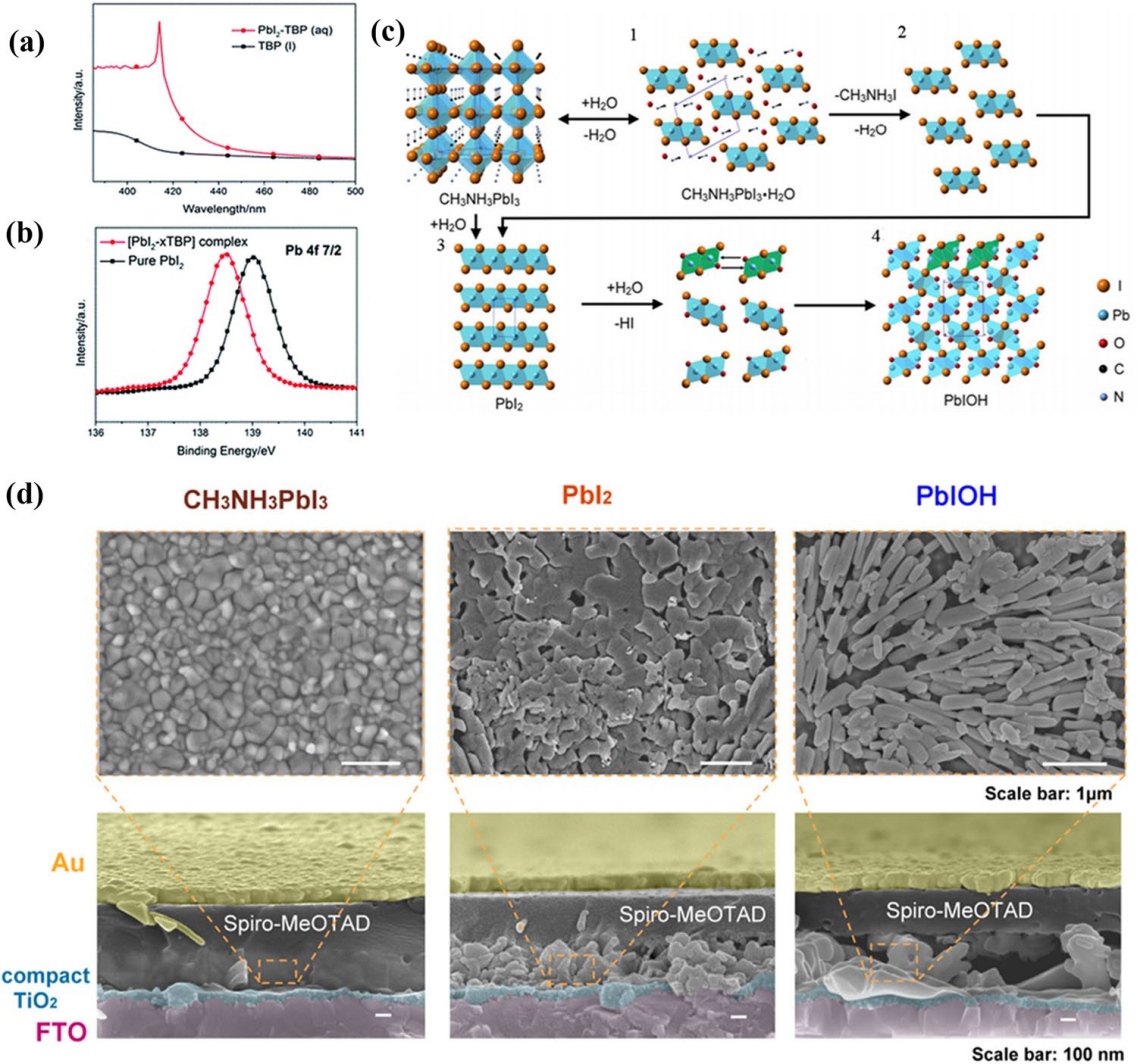
**a** The UV-vis absorption of TBP liquid (dark line) and PbI_2_ solution in TBP (red line). **b** Pb 4f7/2 XPS spectra consuming of PbI_2_. **a**, **b** Reproduced with permission from Ref. [159]. Copyright 2014, Royal Society of Chemistry. **c** Crystal structural evolution from CH_3_NH_3_PbI_3_, CH_3_NH_3_PbI_3_·H_2_O and PbI_2_ to PbIOH. **d** FE-SEM images of top views and the cross-section views of relevant phases CH_3_NH_3_PbI_3_, PbI_2_, and PbIOH formed in lead halide perovskite solar cell at different stages. **c**, **d** Reproduced with permission from Ref. [161]. Copyright 2017, American Chemical Society

Besides, several common additives such as Lithium bis(trifluoromethanesulfonyl)-imide (Li-TFSI) and 4-tert-butylpyridine (TBP) in spiro-OMeTAD can influence the stability of perovskite active layers. Many studies have shown that the hygroscopic nature of Li-TFSI can accelerate the degradation of perovskite in humid conditions [155, 156]. As shown in Fig. 10a and b, TBP can react with PbI_2_ to form new complexes [PbI_2_ $\bullet$ *x*TBP], which disintegrates the perovskite structure and deteriorates the device performance [157, 158]. The reaction can be expressed as follows:

2.2-14 $$ PbI_{2}  + xTBP \to PbI_{2}  \cdot xTBP $$

Chen et al. explored the decomposition of PSCs under operating conditions in the presence of light and H_2_O [159]. The crystal evolution processes are displayed in Fig. 10c. As mentioned in Sect. 2.1.1, MAPbI_3_ can react with H_2_O generating intermediate CH_3_NH_3_PbI_3_·H_2_O in low-humidity conditions [63, 95, 96], which has little effect on the device performance. However, CH_3_NH_3_PbI_3_·H_2_O can further decompose into PbI_2_ once exposed to high humidity, destroying the perovskite structure and reducing the chemical stability of PSCs. H_2_O would further react with axial I of [PbI_6_]^4−^ to form [PbI_4_O_2_]^6−^ under light irradiation or in the presence of spiro-MeOTAD. Given that the ionic radius of I^−^ (2.06 Å) is significantly bigger than that of O^2−^ (1.26 Å) [136], [PbI_4_O_2_]^6−^ is prone to deform. These deformed [PbI_4_O_2_]^6−^ octahedrons further connect with each other to become PbIOH with the coordination structure of [PbI_5_O_3_]^9−^. Figure 10d presents the scanning electron microscopy (SEM) images of PSCs at different degradation stages. It’s worth noting that in addition to H_2_O and light, spiro-MeOTAD is another key factor to stimulate the decomposition of MAPbI_3_ into PbIOH, indicating that spiro-OMeTAD has a crucial influence on the chemical stability of PSCs.

Because of the intrinsic thermal instability of organic HTLs in normal PSCs, a lot of research efforts are devoted to inorganic HTLs [160–162], such as CuSCN, which is a promising candidate due to its low cost and durability [163–165]. It’s reported that the CuSCN can catalyze the thermal degradation process of perovskite films even in the absence of moisture and oxygen [82, 166–168], although the thermal stability of CuSCN itself is excellent. CH_3_NH_3_I and CuSCN can react to form CH_3_NH_3_SCN and CuI as follows:

2.2-15 $$ \left( {CH_{3} NH_{3} } \right)PbI_{3}  + CuSCN\xrightarrow{{heat}}CuI + PbI_{2}  + CH_{3} NH_{3} SCN $$

### Metal Electrodes

Lead halide perovskites not only react with the adjacent layers like charges transport layers, but also experience chemical interactions with the top metal electrodes through ions migrations. The common metal electrodes, such as aluminum (Al), silver (Ag) and gold (Au), can be corroded when in contact with hybrid perovskites or air. These metal ions can diffuse through charge transport layers into perovskites and meanwhile the halide species like I^−^ can migrate to the metal electrode, which result in reactions between metal and halide ions. It has been reported that almost all reactions between metal electrode and perovskites cause severe performance and stability deterioration of PSCs. Therefore, exploring the reactions of the metal electrodes is helpful to have a better understanding of the chemical stability issues of PSCs.

#### Ag Electrode-Induced Reactions

Ag is prone to suffer from corrosion or contamination in contact with lead halide perovskite films. Studies found that I^−^ in perovskites can react with spiro-OMeTAD^+^ at the interface, resulting in a large amount of I^−^ accumulating in the spiro-OMeTAD layer [49, 169]. Strikingly, I^−^ passes through the Spiro-OMeTAD layer and migrates to the Ag contact, meanwhile, Ag ions also diffuse into the perovskite layer from the top electrode, generating AgI in the PSCs [93, 170, 171].

**Fig. 11:**
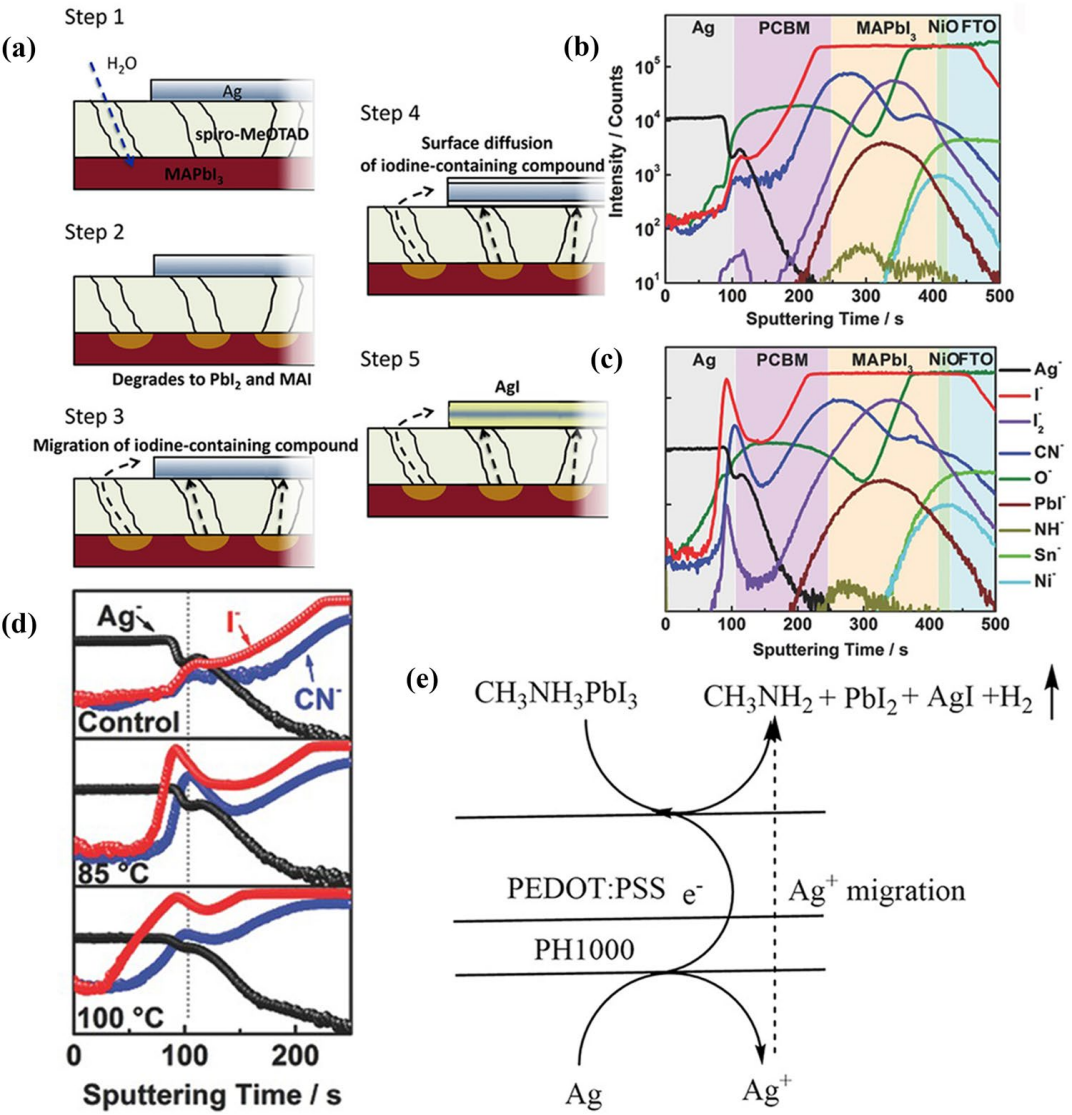
** a** Schematic illustration of a proposed mechanism of AgI formation. Reproduced with permission from Ref. [174]. Copyright 2015, Wiley-VCH. ToF-SIMS elemental depth profiles **b** before and **c** after a thermal treatment at 85 °C for 24 h. **d **The depth profiles of Ag^-^, I^-^ and CN^-^ after different temperature of thermal treatment. **b-d** Reproduced with permission from Ref. [175]. Copyright 2017, Wiley-VCH. **e** The possible reaction mechanism of the PH1000-involved electrochemical corrosion of Ag electrode. Reproduced with permission from Ref. [41]. Copyright 2018, Wiley-VCH

Kato et al*.* reported that moisture can promote the production of AgI and a five-steps mechanism is proposed to understand the observations (Fig. 11a) [171], including (1) diffusion of H_2_O into perovskite through the pinholes in spiro-MeOTAD layer; (2) H_2_O-induced decomposition of MAPbI_3_ and production of volatile species containing I^−^ (e.g., HI); (3) Migration of these volatile species to the bottom or top Ag layer; (4) Surface diffusion of the volatile species containing I^−^; (5) AgI formation. The chemical reaction of AgI formation can be expressed as follows:

2.3-1 $$2HI+2Ag\to 2AgI+{H}_{2}\uparrow $$

In addition, thermal treatment can also accelerate the formation of AgI in inverted PSCs. In Fig. 11b–d, time-of-flight secondary ions mass spectroscopy (ToF–SIMS) tests revealed that thermal treatment triggered a significant accumulation of I^−^, I_2_^−^, and CN^−^ ions at the PCBM/Ag interface, indicating two crucial facts: the decomposition of MAPbI_3_ and the diffusion of both I^−^ and MA^+^ ions [172]. The formation of AgI accelerates the release of MA^+^ and I^−^ ions from the GBs of the perovskite layer and reconstructs the grain domains, leading to more defects both in the perovskite films and at the interface. Consequently, the PSCs suffer from degraded performance due to the formation of AgI.

Ma et al*.* reported electrochemical corrosion of Ag grid electrodes by PEDOT:PSS in flexible PSCs, which is a major reason for the low performance and poor chemical stability of the devices [41]. This redox reaction involves the reduction of the highly conductive PEDOT:PSS layer and the oxidation of the Ag electrode, which is proposed to be three steps including (Fig. 11e): (1) Ag electrode easily loses an electron to form Ag^+^ with the aid of acidic PEDOT:PSS layer; (2) PEDOT:PSS layer receives an electron and is reduced; (3) Ag^+^ enters into the perovskite precursor solution and reacts with I^−^ to generate AgI.

Therefore, regardless of the structure of PSCs, the corrosion of Ag electrode is usually ascribed to the chemical reactions between Ag and the components of perovskites, which is achieved through Ag^+^ migration or halide ions diffusion [173–175]. Eventually the formed AgI impedes the charge transport and reduces the photovoltaic performance and chemical stability of PSCs.

#### Au Electrode-Induced Reactions

Au, an inert metal and a common electrode material [5, 176], has been found to react with lead hybrid perovskite in previous studies [177–181]. Researches show that Au diffuses from the electrode across the HTL into the perovskite layer under certain conditions such as light soaking [182] or heat aging [183], resulting in an irreversible loss in photovoltaic performance and chemical stability of PSCs. Tarasov et al*.* proposed the corrosion mechanism of Au electrode based on its reaction with iodine-based perovskites [184]. Generally, the intensive UV irradiation of perovskite would induce the release of volatile I_2_ and MAI, forming highly reactive polyiodide melts with a general formula of MAI-nI_2_. This MAI-nI_2_ could react strongly with Au at room temperature and form [AuI_2_]^−^ and [AuI_4_]^−^ complexes. Consequently, a new (MA)_2_Au_2_I_6_ phase is observed on the Au interface, which is detrimental to the performance of PSCs.

2.3-2 $$ 2MAI - nI_{2}  + 2Au\xrightarrow{{light}}\left( {MA} \right)_{2} Au_{2} I_{6}  $$

**Fig. 12:**
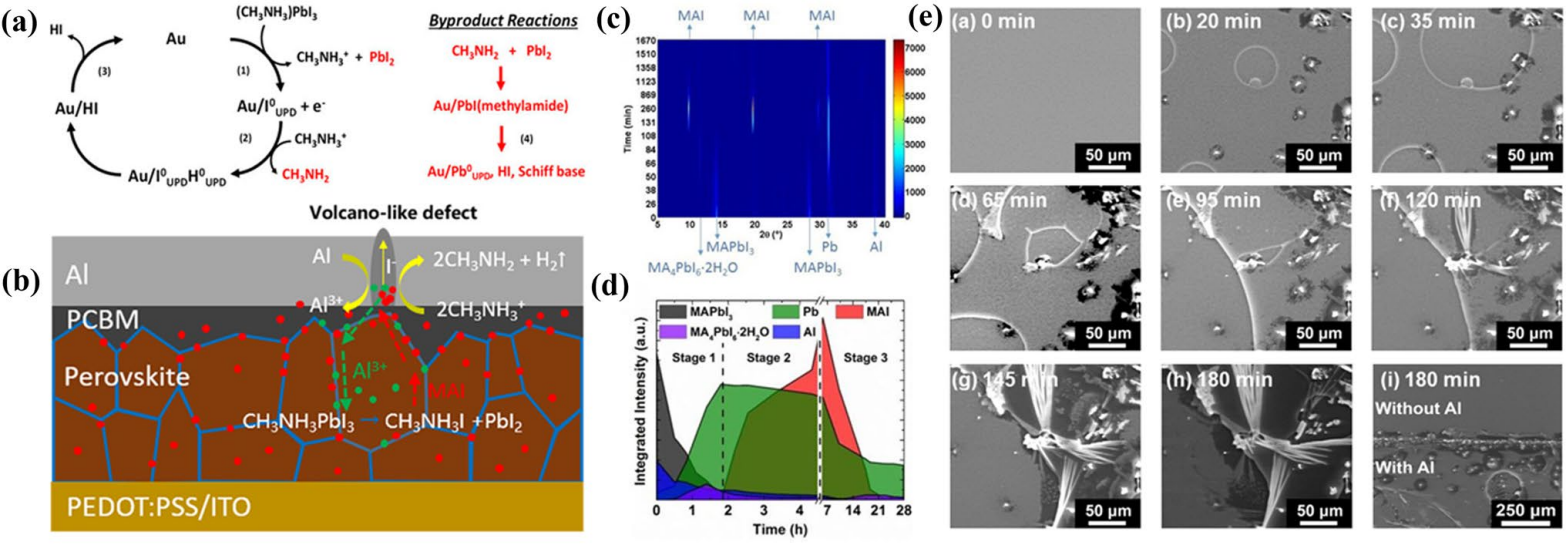
**a** Proposed noble metal catalyzed degradation at MAPbI_3_/Au heterojunctions where the byproducts methylamine and PbI_2_ (in red) lead to the eventual formation of Pb^0^. Reproduced with permission from Ref. [188]. Copyright 2019, American Institute of Physics. **b** Proposed chemical corrosion of Al electrode of p-i-n type PSCs by the diffusion of CH_3_NH_3_I. Reproduced with permission from Ref. [192]. Copyright 2020, Wiley-VCH. In situ XRD analysis of the degradation of the LED stack with moist air in the dark: **c** In situ XRD spectra versus time. **d** Integrated XRD peak intensities of key materials versus time. **e** In situ SEM analysis on the LED stack in an environmental chamber at various time points from 0 to 180 min; the last one, SEM image near the boundary of the Al area after 180 min exposure showing that only the area covered with Al undergoes H_2_O-mediated decomposition. **c-e** Reproduced with permission from Ref. [193]. Copyright 2016, American Chemical Society

In addition, MAPbI_3_/Au interface can catalyze the perovskite degradation reaction. A type of reduction/oxidation reaction termed underpotential deposition (UPD) involving lead, iodine, and hydrogen can occur at the MAPbI_3_/Au interface [185]. UPD is a surface adsorption reaction that changes the oxidation state of ions, and occurs spontaneously at a lower voltage than the bulk reaction potential. XPS measurements demonstrate that Pb^0^_UPD_ and I^0^_UPD_ form at MAPbI_3_/Au surface, and the whole degradation pathway of MAPbI_3_/Au can be described with the following steps (Fig. 12a): (1) I^−^ loses an electron to form I^0^_UPD_ that absorbs on the Au surface (Eq. 2.3-3), which induces the decomposition of MAPbI_3_ into PbI_2_ and CH_3_NH_3_^+^. (2) CH_3_NH_3_^+^ captures a free electron to form methylamine gas and H^0^_UPD_ simultaneously (Eq. 2.3-4). (3) The adsorbed I^0^_UPD_ reacts with H^0^_UPD_ to generate HI, I_2_, or H_2_ gases (Eq. 2.3-5, 2.3-6, 2.2-7). (4) The byproduct CH_3_NH_2_ reacts with PbI_2_ via PbI (CH_3_NH_2_) interphase to form more HI, imines, and Pb^0^_UPD_ (Eq. 2.3-8). Eventually, the degradation reaction of MAPbI_3_ will stop when the surface of the Au catalyst is completely covered with Pb^0^_UPD_. Hence, the detrimental interfacial chemical reactions provide a source for defects and reduce the chemical stability of PSCs.

2.3-3 $$ I^{ - } \xrightarrow{{Au}}I_{{UPD}}^{0}  + e^{ - }  $$

2.3-4 $$ CH_{3} NH_{3}^{ + }  + e^{ - } \xrightarrow{{Au}}CH_{3} NH_{2}  \uparrow  + H_{{UPD}}^{0}  $$

2.3-5 $${I}_{UPD}^{0}+{H}_{UPD}^{0}\to HI\uparrow $$

2.3-6 $${2I}_{UPD}^{0}\to {I}_{2}$$

2.3-7 $${2H}_{UPD}^{0}\to {H}_{2}\uparrow $$

2.3-8 $$C{H}_{3}N{H}_{2}+Pb{I}_{2}\stackrel{Au}{\to }C{H}_{2}=NH\uparrow +{Pb}_{UPD}^{0}+2HI\uparrow $$

Notably, Au not only reacts with perovskite through ions migrations but also can be corroded by interaction with the inorganic holes transport material CuSCN. It has been reported that the reactive thiocyanate anions are prone to react with Au electrode under an electrical bias, forming an undesired potential barrier for charge carriers [153, 165, 186, 187], which may seriously affect the device performance and chemical stability.

#### Al Electrode-Induced Reactions

Although Al is commonly employed as the top electrode in inverted PSCs, Al is chemically unstable due to its easy oxidation by air and reactions with lead halide perovskites [171, 188]. For example, Ma et al*.* found that both CH_3_NH_3_^+^ and I^−^ can migrate towards the Al electrode through a PC_61_BM layer, resulting in the chemical corrosion of the Al electrode and the degradation of the perovskite film [189]. In particular, CH_3_NH_3_I is highly acidic and can react with Al as shown below:

2.3-9 $$ 6CH_{3} NH_{3} I + 2Al \to 6CH_{3} NH_{2}  \uparrow  + 2AlI_{3}  + 3H_{2}  \uparrow   $$

For the overall degradation of PSC, the chemical corrosion of Al electrode can be described in Eq. 2.3-10. The reaction process involves the following steps (Fig. 12b) including: (1) formation of CH_3_NH_3_I from perovskite decomposition, (2) diffusion of CH_3_NH_3_I through the thin PC_61_BM region, (3) reaction between CH_3_NH_3_I and Al, yielding AlI_3_, CH_3_NH_2_ and H_2_, (4) formation of bubble and volcano-like surface defects owing to the release of H_2_ gas, (5) further decomposition of perovskite due to the Al^3+^ diffusion into perovskite layer.

2.3-10 $$6(C{H}_{3}{NH}_{3})Pb{I}_{3}+2Al\to 2Al{I}_{3}+{6PbI}_{2}+{6CH}_{3}{NH}_{2}\uparrow +{3H}_{2}\uparrow $$

To further investigate the Al-perovskite reactions, Zhao et al*.* employed in situ X-ray diffraction (XRD) analyses (Fig. 12c and d) and SEM measurements (Fig. 12e) to study the redox reactions between the Al electrode and perovskite [190]. The chemical degradation process can be divided into three stages. Stage 1: Al reacts with a perovskite in the presence of moisture, resulting in the reduction of Pb^2+^ to Pb^0^ and the crystal nucleation of MA_4_PbI_6_·2H_2_O, which can be expressed as follows:

2.3-11 $$ 2Al + 4\left( {CH_{3} NH_{3} } \right)PbI_{3}  + 2H_{2} O \to \left( {CH_{3} NH_{3} } \right)_{4} PbI_{6}  \cdot 2H_{2} O + 3Pb^{0}  + 2Al^{{3 + }}  + 6I^{ - }  $$

Stage 2: with the continuous redox reaction between Al and Pb^2+^, the further loss of Pb^2+^ in MA_4_PbI_6_·2H_2_O will lead to the formation of MAI as shown below:

2.3-12 $$ 2Al + 3\left( {CH_{3} NH_{3} } \right)_{4} PbI_{6}  \cdot 2H_{2} O \to 12CH_{3} NH_{3} I + 3Pb^{0}  + 2Al^{{3 + }}  + 6I^{ - }  + 2H_{2} O $$

Stage 3: Once metal Al is completely oxidized to Al^3+^, further exposure to moisture could cause Pb^0^ to reoxidize and hydrate forming PbO·xH_2_O. In addition, volatile MAI gradually disappears due to prolonged exposure to air. Here, moisture not only promotes ions diffusion but also keeps the reactions between Al and MAPbI_3_ forward.

## Strategies for Improving the Chemical Stability

Interfacial chemical reaction is a key factor affecting the chemical stability of lead halide PSCs. According to the above discussion, not all the interfacial reactions in PSCs are detrimental to device performance and chemical stability, such as the reactions at SnO_x_-Cl/perovskite interface. Therefore, in order to improve the chemical stability of PSCs, it’s necessary to inhibit unfavorable interfacial reactions. Two effective strategies have been proposed to reduce adverse interfacial reactions in the past few years. One is to insert a buffer layer at the contact interface to block ions migration or the invasion of water and oxygen. At present, many materials, such as PMMA [167], Al_2_O_3_ [82], and Cr_2_O_3_ [181], are introduced to optimize contact interfaces as a buffer layer. Another approach is to employ additives, such as 2-amylpyridine [158] and ionic liquids [191], in perovskites to reduce the chemical activity of the reactants.

### Buffer Layers

Various buffer layers have been developed to modify the perovskite surface to prohibit the chemical reactions with various materials, including air, NiO_x_, spiro-OMeTAD, CuSCN, TiO_2_, ZnO, metal electrode and so on, based on different reaction mechanisms. The effective approaches for these interface modifications are detailed as follows.

**Fig. 13:**
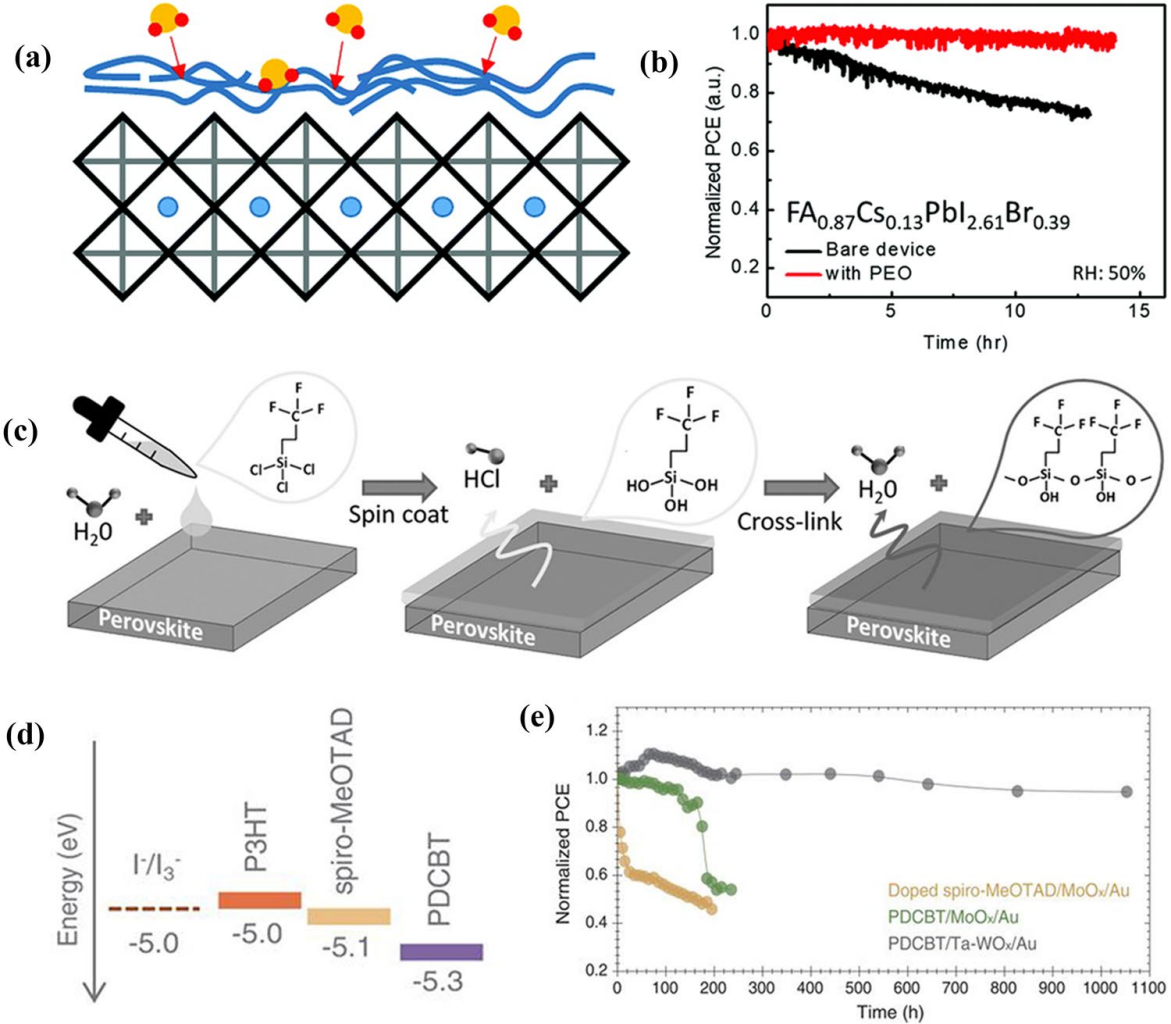
**a** Schematic illustration of polymer PEO thin film assembled on the perovskite structure.** b** Operational stability test for evolution of PCE of FA_0.87_Cs_0.13_Pb(I_0.87_Br_0.13_)_3_ devices without and with PEO, continuously measured under xenon-lamp simulated fullspectrum AM 1.5, 100 mW cm^-2^ equivalent irradiance in air (humidity ~50 RH%) without any ultraviolet filter, held at maximum power point during ageing, and tested at 0.03-s time intervals.** a**,** b** Reproduced with permission from Ref. [195]. Copyright 2018, Royal Society of Chemistry. **c** Schematic diagram showing the cross-link process of fluoro-silane layer on the perovskite film. Reproduced with permission from Ref. [196]. Copyright 2016, Wiley-VCH. **d** The corresponding redox potential of I^-^/I_3_^-^and the homo levels of P3HT, PDCBT, and spiro-MeOTAD. **e** The unencapsulated device photostability tests under continuous one sun illumination in a home-built chamber filled with N_2_. **d, e** Reproduced with permission from Ref. [199]. Copyright 2017, AAAS

*Air/perovskite interface*: The buffer layer, especially polymer materials, can isolate moisture or oxygen and inhibit the degradation of lead halide perovskite films. For example, hygroscopic polymer poly(ethylene oxide) (PEO) can chemically interact with undercoordinated Pb ions on a perovskite surface, passivating defect sites and reducing charge recombination loss [192]. In Fig. 13a and b, PEO prevents surface hydration reaction of perovskite by absorbing H_2_O before perovskites do, leading to greatly improved chemical stability of PSCs. A number of hydrophobic polymers, such as polystyrene [193], PTzDPPBTz [91] and carbon-based materials [194], are also used to protect perovskites from H_2_O. In Fig. 13c, Trichloro(3,3,3-trifl uoropropyl)silane was introduced between a perovskite layer and C_60_ ETL, which reacts with a tiny amount of H_2_O existing in the perovskite film to form silanols [193]. Then these silanols can automatically cross-link through forming Si–O–Si (siloxane) bonds to make the insulating layer protect the underlying perovskite film from water erosion.

It has been reported that low-dimensional materials covering perovskite surfaces can effectively insulate O_2_ and improve the perovskite film quality [58]. For example, Kanatzidis et al. inserted a 1D thiazole ammonium lead iodide (TAPbI_3_) interfacial layer between the perovskite and HTL, which serves to passivate the three-dimensional (3D) perovskite film and prevent oxygen invasion [195], leading to an improvement of device efficiency as well as enhancement of chemical stability.

*NiO*_*x*_*/perovskite interface:* The interfacial redox reaction between Ni^3+^ in NiO_x_ and MAI salt will generate PbI_2_-rich holes extraction barriers, which can lower *V*_oc_ and accelerate perovskite degradation by deprotonating precursor amine and oxidizing iodide to interstitial iodine [148]. A modifier layer, trimethylolpropane tris(2-methyl-1-aziridinepropionate) (SaC-100), was used to modify the NiO_x_/MAPbI_3_ contact interface [83]. Results reveal that N and O atoms in SaC-100 can passivate the uncoordinated Ni^3+^ and Pb^2+^ defects in NiO_x_ and MAPbI_3_ through producing Lewis adducts, respectively. In addition, the reaction between NiO_x_ and MAPbI_3_ is suppressed in the presence of SaC-100, which is helpful for improving the conductivity of NiO_x_ and inhibiting the degradation of MAPbI_3_ films.

*Spiro-OMeTAD/perovskite interface*: Buffer layer materials can not only resist the invasion of air, but also block the undesired contacts between adjacent layers. For example, Wang et al*.* reported an effective buffer layer montmorillonite (MMT), which was inserted between perovskite and spiro-OMeTAD to prevent TBP from reacting with PbI_2_, because MMT can interact with TBP and build its intercalated structure by hydrogen bond. Consequently, the efficiency and chemical stability of PSCs are largely improved simultaneously [157]. I_2_ deriving from perovskite tends to react with HTLs, such as Spiro-OMeTAD, whose HOMO level is close to the oxidation potential of I^−^/I_3_^−^ (~ − 5.0 eV). In Fig. 13d, depositing a polythiophene derivative with a lower HOMO level approaching − 5.3 eV on the perovskite surface can effectively suppress I^−^/I_3_^−^ reaction, resulting in PSCs with enhanced efficiency and stability (Fig. 13e) [196].

*CuSCN/perovskite interface:* In order to promote the chemical stability of PSCs with CuSCN HTLs, it’s necessary to insert a buffer layer between perovskite and CuSCN. Snaith et al*.* not only introduced mesoporous Al_2_O_3_ at the perovskite/CuSCN interface to reduce the contact area of CuSCN with perovskite, but also encapsulated the complete cell with PMMA to prevent degradation products from release [82]. It has been found that if an electrical bias is applied on the PSC, an electrical potential-induced reaction between Au and SCN^−^ would occur, leading to poor operational stability of PSCs. Therefore, a thin reduced graphene oxide (rGO) interlayer was introduced between CuSCN and Au electrode to migrate the degradation reaction [165]. In addition, Cu_2_O [197] and Cs:NiO_x_ [198] interfacial layers were also used to reduce these detrimental interfacial chemical reaction for PSCs.

**Fig. 14:**
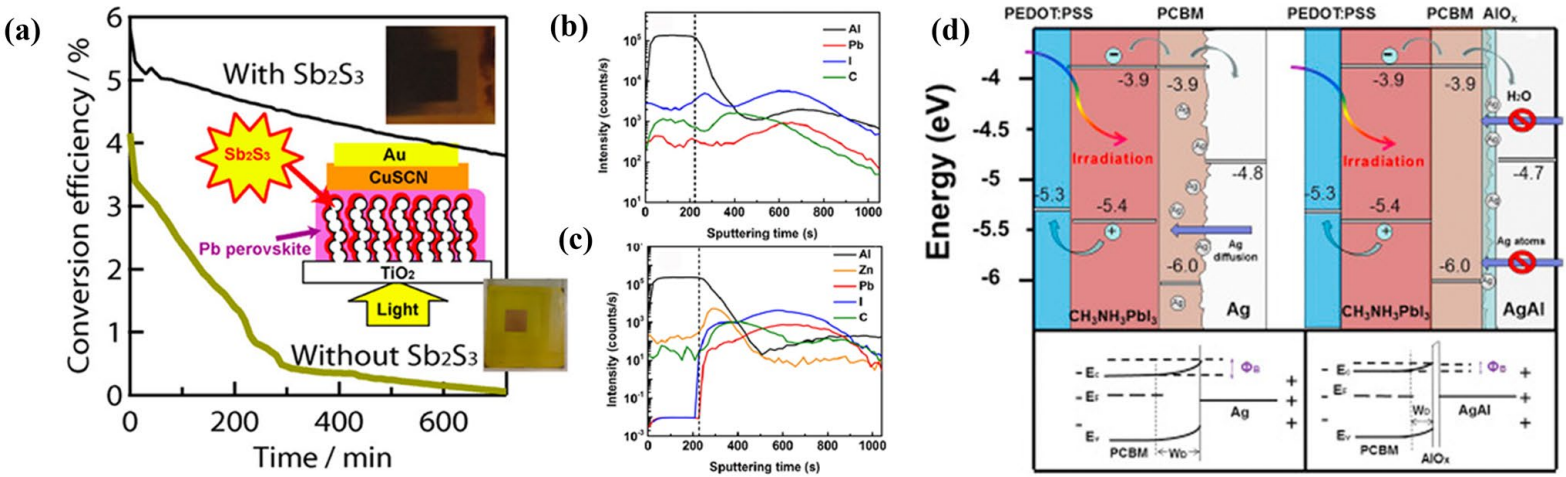
**a** Variation of photoenergy conversion efficiencies of solar cells during light exposure (AM1.5, 100 mW cm^-2^) without encapsulation in air for 12 h. Reproduced with permission from Ref. [125]. Copyright 2014, American Chemical Society. Composition depth profiles of the perovskite devices by SIMS: **b** with only PCBM as ETL; **c** with PCBM/ZnO bilayer as ETL. **b, c** Reproduced with permission from Ref. [204]. Copyright 2015, Elsevier. **d** Schematic energy level diagrams of cells with PCBM/Ag and PCBM/AgAl. Reproduced with permission from Ref. [206]. Copyright 2016, Elsevier. (Color figure online)

*TiO*_*2*_*/perovskite interface*: Ito et al*.* performed light irradiation tests of PSCs with/without a Sb_2_S_3_ inserted layer between TiO_2_ and MAPbI_3_ [124]. For the device without Sb_2_S_3_, the black MAPbI_3_ layers completely decompose to yellow PbI_2_ by losing HI and CH_3_NH_2_. On the other hand, the device with Sb_2_S_3_ can deactivate the reaction of I^−^/I_2_ at the surface of TiO_2_, so the MAPbI_3_ layer is stable and durable against light exposure as shown in Fig. 14a.

*ZnO/perovskite interface*: A thin layer of MgO and protonated ethanolamine were in sequence deposited on the ZnO surface to resolve the poor chemical stability of ZnO-based device [199]. Moreover, 1,2-ethanedithiol [200], poly(ethylenimine) [132] and graphene oxide quantum dots [126] can also play the same role.

*Metal/perovskite interface*: The chemical instability of PSCs induced by metal electrode corrosion can be suppressed by interfacial modifications. I^−^ in the perovskite can migrate to an Al electrode through PCBM layer to react with Al, forming aluminum iodide at the Al/perovskite interface. A bathocuproine (BCP) buffer layer has been reported to insert the interface between the Al electrode and PC_61_BM [189]. Test results support that this buffer layer can block the CH_3_NH_3_I migration and hence prevent the Al electrode from chemical corrosion by CH_3_NH_3_I, improving the chemical stability of the PSCs. Qiu et al*.* introduced a ZnO layer between PCBM and an Al electrode to prevent perovskite from reacting with Al, ToF–SIMS characterizations demonstrated that ZnO layer can effectively inhibit the diffusion of I^−^ ions (Fig. 14b and c) [201].

**Fig. 15:**
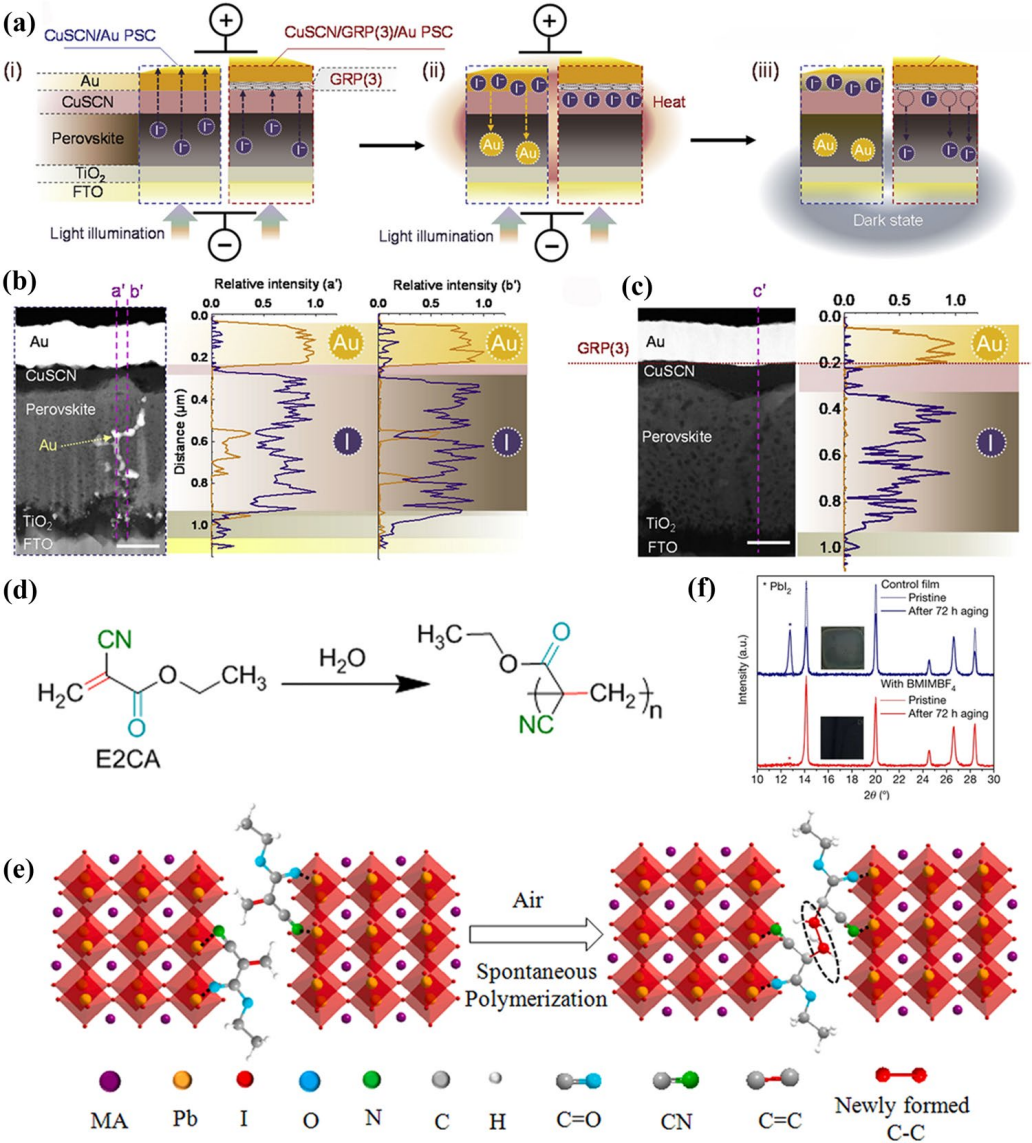
The iodide ion (I^-^) and Au diffusion in CuSCN/Au and CuSCN/GRP (3)/Au PSCs. **a** Schematic of the diffusion process during the light illumination under an applied electrical bias and dark state without the bias. Cross-sectional STEM images of CuSCN/Au** b** and CuSCN/GRP (3)/Au PCSs **c** after 3 cycles of continuous light illumination (12 h) and dark state (12 h). The scale bar is 200 nm. The EDX scan (I and Au) along the vertical lines (a´, b´, and c´) are also provided. **a-c** Reproduced with permission from Ref. [208]. Copyright 2020, Elsevier. **d** Chemical structure and moisture induced polymerization of E2CA with marked functional groups: C=C (red), C≡N (olive) and C=O (cyan). **e** Schematic illustrations of spontaneous grain polymerization in MAPbI_3_-E2CA films. E2CA chemically anchors to GBs with C=O and C≡N groups and spontaneously polymerizes to a polymer at GBs when exposed in moisture air. **d**, **e** Reproduced with permission from Ref. [214]. Copyright 2019, Elsevier.** f** XRD patterns of pristine and aged samples of control film and film containing BMIMBF_4_ (0.3 mol%) on NiO/FTOcoated glass substrates. The stars represent the decomposition product of PbI_2_ in the films. The insets show images of the aged samples (around 2.8 cm × 2.8 cm) after 72 h of light-soaking at 60-65 °C. Reproduced with permission from Ref. [217]. Copyright 2019, Springer Nature. (Color figure online)

Cr/Cr_2_O_3_ interlayer provides a buffer to shield top Ag electrodes from chemical corrosion caused by HI liberated from the MAPbI_3_ degradation [181, 202]. Compared with pure Ag, an AgAl alloy electrode shows much higher stability in aging tests, which is related to AlO_x_ formed at the PCBM/AgAl interface during thermal evaporation and aging [203]. In Fig. 14d, this AlO_x_ interlayer can suppress the migration of Ag atoms to the active layer, strengthen the metal contact with PCBM and prevent moisture encroachment. In addition, nanostructured carbon layers, including N-doped graphene, PCBM and carbon quantum dots, were inserted between a perovskite layer and an Ag electrode to block I^−^ and Ag diffusion [204]. The degradation reaction of perovskite by ions migration was ultimately inhibited, and thus the chemical stability of PSCs was greatly enhanced. Moreover, a graphene barrier between CuSCN and Au can inhibit I^−^ migration and perfectly block Au diffusion, as shown in Fig. 15a–c [205], successfully restraining undesired chemical reactions between I^−^ and the Au electrode. Many groups introduced stable buffer layers through interfacial reactions to reduce ions migration [206]. For example, hexamethyldisilathiane is deposited on a perovskite surface to react with Pb^2+^ and form stable PbS buffer layer, which can effectively suppress I^−^ diffusion and prevent corrosion of metal electrodes [207].

### Additives Engineering

*Water-induced reactions*: Various additives have been demonstrated to change the physical or/and chemical properties of metal halide perovskites and thus improve the moisture-/oxygen-/light- stability of the materials [208]. For example, a fluorinated additive named 1,1,1-trifluoro-ethylammonium iodide (FEAI) was introduced into MAPbI_3_ to reduce the moisture-induced degradation reactions, because the hydrophobic CF_3_-terminal group on the perovskite surface resist the invasion of moisture [209]. Zhao et al*.* employed hygroscopic polyethylene glycol (PEG) scaffold to fabricate moisture-stable perovskite films because the omnipresent PEG molecules can absorb H_2_O efficiently. The resulting compact moisture barrier around perovskite crystal grains can prevent water from penetrating into the films [210]. The additive ethyl 2-cyanoacrylate (E2CA) can spontaneously polymerize into a compact polymer once exposed to moisture as shown in Fig. 15d and e, blocking the penetration channels of moisture due to its hydrophobic nature [211]. Moreover, poly(4-vinylpyridine) (PVP) [212], poly(ethylene oxide) (PEO) [192], and trimethylolpropane triacrylate (TMTA) [213] can also be used as additives to reduce the reactions between perovskite and H_2_O.

*Oxygen-induced reactions*: Snaith et al*.* introduced the ionic liquid 1-butyl-3-methylimidazolium tetrafluoroborate (BMIMBF4) into perovskite precursor and found that [BMIM]^+^ cations were bound to the surface sites, hence suppressing the surface degradation reactions induced by oxygen or moisture adsorption (Fig. 15f) [214]. The thiourea has been demonstrated to form Pb–S bonds on the outermost layer of perovskite, which efficiently suppressed the absorption of oxygen and prevented the formation of superoxide [215].

*Light-induced reactions*: The solid ionic additive 1-butyl-1-methylpiperidinium tetrafluoroborate ([BMP]^+^[BF_4_]^−^) was adopted to enhance the photostability of PSCs under full-spectrum sunlight [191]. It’s reported that the generation of I_2_ under illumination is one of the main factors causing the photo-degradation of perovskites [216]. The interstitial I^−^ ions capture the holes to become neutral interstitial iodine atoms, and these neutral atoms need to diffuse and combine to produce I_2_ [112]. [BMP]^+^[BF_4_]^−^ therefore suppressed the photochemical reactions of perovskite by reducing the surface defects, such as interstitial pairs and iodide vacancies, and retarding diffusion of neutral iodine interstitials.

*CTL-induced reactions*: The additives can also inhibit undesirable reactions occurring at charges transport layer/perovskite interfaces. Han et al*.* used a new additive 2-amylpyridine (2-Py) to replace TBP in Spiro-OMeTAD and found that the corrosion of perovskite was suppressed [158]. The existence of the amyl chain at the ortho-position of pyridine in 2-Py is vital to restrain the perovskite degradation reaction, which is attributed to the following aspects: 1) the amyl chain near the nitrogen atom can reduce the coordination ability of pyridine; 2) the steric hindrance from the o-substitution of pyridine prevents it from penetrating the perovskite. In addition, 2-Py can increase the dissolution and dispersion of Li-TFSI compared with TBP, which has a positive effect on the conductivity of the Spiro-OMeTAD HTL. Other additives, like TiO_2_ nanoparticles (NPs)-modified CNT (CNT:TiO_2_) [217], graphene oxide (GO) [218], and 1,6-diazidohexane (N3) [219] can also be introduced into spiro-OMeTAD to minimize the TBP-PbI_2_ complex formation and hence improve the chemical stability of the perovskite/HTL interface.

**Fig. 16:**
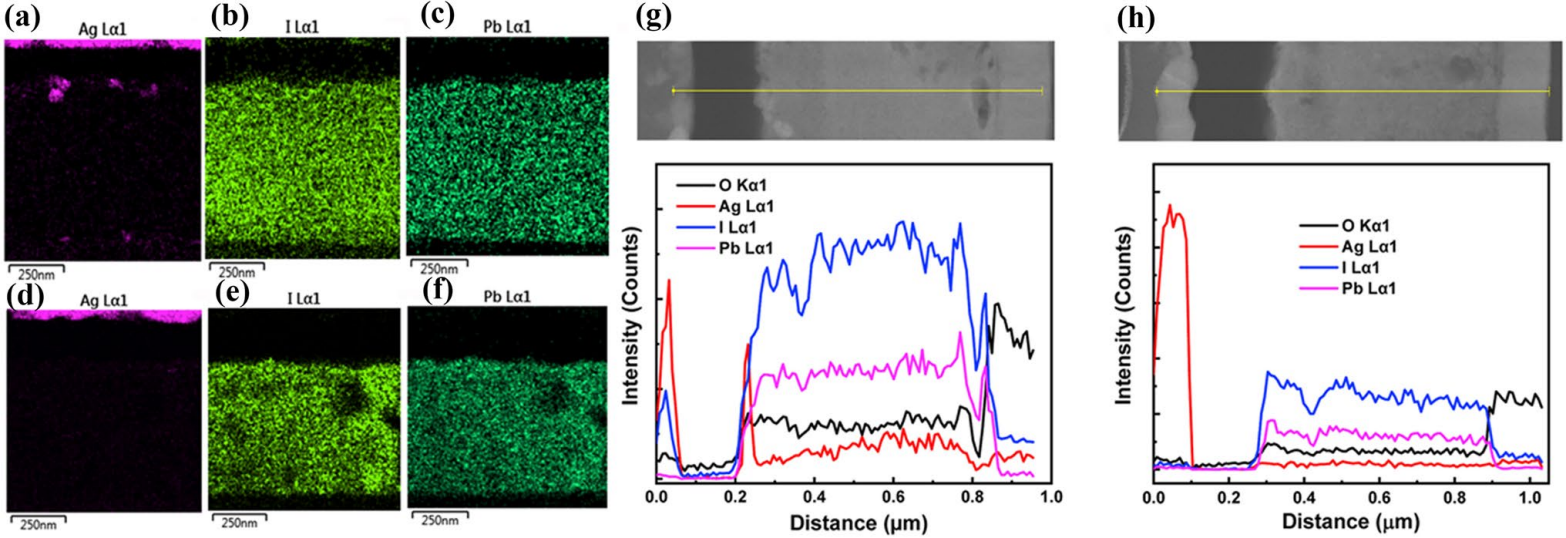
** a-f** Energy-dispersive X-ray spectra (EDX) mapping of the aged pure PVSK device **a **Ag,** b** I, and **c** Pb, and the aged caffeine-containing PVSK device **d** Ag, **e** I, and **f** Pb. **g**, **h **EDX line scans of **g** aged pure PVSK device and **h** aged caffeine-containing PVSK device. Reproduced with permission from Ref. [224]. Copyright 2019, Elsevier. (Color figure online)

*Metal electrode-induced reactions*: It has been reported that the ions migration can initiate the degradation of devices [113, 220], which can be efficiently inhibited by some additives with special functional groups. For example, caffeine with two conjugated carboxyl groups interacts strongly with Pb^2+^ ions to slow down the perovskite crystal growth and thus produce high-quality films [221]. In Fig. 16, the Energy-dispersive X-ray spectra (EDX) mapping shows that Ag can diffuse into the whole perovskite region and I^−^ ions also migrate through the PTAA layer in the control device, while there is no obvious indication of such similar ions migrations in the caffeine-incorporated device. The caffeine significantly suppresses ions migration, and hence the chemical stability of PSCs is effectively enhanced. Chen et al. employed the methimazole (MMI) to form the MMI-PbI_2_ complex in situ at the GBs, and these surface patches can also effectively suppress Ag diffusion and simultaneously retard I^−^ migration [222].

The multifunctional additive of dipentaerythritol pentaacrylate (DPPA) can impede I^−^ movements due to synergistic effects of its functional groups [223]. The CH_2_=CH groups in DPPA crosslink at GBs and block the channels of ions migrations. And the -OH groups in DPPA can bond with I^−^ in the perovskite through hydrogen bond interaction, chemically immobilizing these migrated I^−^. In addition, the polystyrene (PS) [224] and PCBM [74] can also obstruct ions migrations across GBs and dissociated ions can only move around their original sites. Therefore, the problem of chemical stability induced by ions migration can be well solved.

## Conclusions and Outlook

Although lead halide PSCs have already achieved a high certified PCE up to 25.7% through a low-cost and simple fabrication process, the long-term stability still lags far behind the commercial application standard. Therefore, the top priority is to explore the degradation mechanism of lead halide PSCs in more depth, which is helpful for achieving high-efficiency and stable devices. As one of the most important factors influencing the photovoltaic performance and chemical stability of PSCs, interfacial chemical reactions due to environment conditions, interface contacts and metal electrode are systematically elucidated in this paper. Their reaction mechanisms as well as influence on interface defects, non-radiative recombination and degradation process are summarized. Up to now, there is no unified theory that generalizes all interface reactions. For example, lead halide perovskites degrade easily to form PbI_2_ and other lead-containing compounds once exposed to humid environment. O_2_ capture photo-induced electrons to generate superoxide O_2_^·−^ with the aid of light, which can induce quick degradation of lead halide perovskite films. In addition, some chemical reactions at charge transport layers/perovskite interface can produce undesirable interfacial defects and reduce charge transport capacity of ETL or HTL, eventually deteriorate the chemical stability and photovoltaic performance of PSCs. It’s noteworthy that Cl-containing SnO_2_ ETL can react friendly with lead halide perovskites to passivate the interface defects and improve the device efficiency. The interfacial reactions are induced by ions migrations, for example, I^−^ can diffuse into a metal electrode and simultaneously metal ions can also migrate through the perovskite, which corrode the metal electrode and decrease the operational lifetime of PSCs.

At present, various buffer layers or additives have been employed to suppress the detrimental interfacial reactions and optimize the device performance as well as long-term stability. Therefore, it’s necessary to investigate the chemical properties of additives or buffer layer materials in depth, because their different functional groups may play completely different roles in the photovoltaic performance. The fluorine-containing additives can improve the hydrophobic properties of lead halide perovskite films, and effectively reduce the humidity-induced degradation of PSCs. The detrimental I_2_ in the lead halide perovskite easily induces the shallow charge traps and accelerates the light-decomposition of perovskite. The hydrazine functional group can reduce these I_2_ in perovskite precursor solutions to I^−^, which significantly improve the light stability of perovskites. In addition, more attentions should be paid to benign chemical reaction in PSCs and thus we can make use of these reactions to further improve the efficiency and stability of PSCs. For example, the organic long chain amine (e.g. phenylethylamine, butylamine) can react with PbI_2_ to form stable 2D perovskite on the bulk perovskite surface, contributing to the decreased non-radiative recombination and enhanced water resistance of 3D perovskite films. Investigating novel and stable charge transport layer and electrode materials is also of significance for the chemical stability of PSCs. Future molecular design of charge transport materials should consider the energy level alignment, high carrier mobility and stability. The cheap and chemically stable carbon electrode might be an alternative to reduce the cost and deterioration for lead halide PSCs. It’s expected that rational management of interfacial chemical reactions in the whole PSCs will lead to substantial performance enhancement, paying the way for the commercialization of PSCs in the future.

## References

[1] Kojima A, Teshima K, Shirai Y, Miyasaka T (2009). Organometal halide perovskites as visible-light sensitizers for photovoltaic cells. J. Am. Chem. Soc..

[2] Im J-H, Lee C-R, Lee J-W, Park S-W, Park N-G (2011). 6.5% efficient perovskite quantum-dot-sensitized solar cell. Nanoscale.

[3] Lee MM, Teuscher J, Miyasaka T, Murakami TN, Snaith HJ (2012). Efficient hybrid solar cells based on meso-superstructured organometal halide perovskites. Science.

[4] Heo JH, Im SH, Noh JH, Mandal TN, Lim CS (2013). Efficient inorganic-organic hybrid heterojunction solar cells containing perovskite compound and polymeric hole conductors. Nat. Photonics.

[5] Min H, Lee DY, Kim J, Kim G, Lee KS (2021). Perovskite solar cells with atomically coherent interlayers on SnO_2_ electrodes. Nature.

[6] Kim H-S, Lee C-R, Im J-H, Lee K-B, Moehl T (2012). Lead iodide perovskite sensitized all-solid-state submicron thin film mesoscopic solar cell with efficiency exceeding 9%. Sci. Rep..

[7] Jiang C, Zhou J, Li H, Tan L, Li M (2022). Double layer composite electrode strategy for efficient perovskite solar cells with excellent reverse-bias stability. Nano-Micro Lett..

[8] Travis W, Glover ENK, Bronstein H, Scanlon DO, Palgrave RG (2016). On the application of the tolerance factor to inorganic and hybrid halide perovskites: a revised system. Chem. Sci..

[9] Qiu L, He S, Ono LK, Qi Y (2020). Progress of surface science studies on ABX_3_-based metal halide perovskite solar cells. Adv. Energy Mater..

[10] Anaya M, Lozano G, Calvo ME, Míguez H (2017). ABX_3_ perovskites for tandem solar cells. Joule.

[11] Yi C, Luo J, Meloni S, Boziki A, Ashari-Astani N (2016). Entropic stabilization of mixed A-cation ABX_3_ metal halide perovskites for high performance perovskite solar cells. Energy Environ. Sci..

[12] Wang S, Sakurai T, Wen W, Qi Y (2018). Energy level alignment at interfaces in metal halide perovskite solar cells. Adv. Mater. Interfaces.

[13] Meng L, You J, Guo T-F, Yang Y (2016). Recent advances in the inverted planar structure of perovskite solar cells. Acc. Chem. Res..

[14] Liu Z, Zhu A, Cai F, Tao L, Zhou Y (2017). Nickel oxide nanoparticles for efficient hole transport in p-i-n and n-i-p perovskite solar cells. J. Mater. Chem. A.

[15] Momblona C, Gil-Escrig L, Bandiello E, Hutter EM, Sessolo M (2016). Efficient vacuum deposited p-i-n and n-i-p perovskite solar cells employing doped charge transport layers. Energy Environ. Sci..

[16] Zhang Z, Liang J, Wang J, Zheng Y, Wu X (2022). Resolving mixed intermediate phases in methylammonium-free Sn–Pb alloyed perovskites for high-performance solar cells. Nano-Micro Lett..

[17] Polman A, Knight M, Garnett EC, Ehrler B, Sinke WC (2016). Photovoltaic materials: present efficiencies and future challenges. Science.

[18] Huang F, Li M, Siffalovic P, Cao G, Tian J (2019). From scalable solution fabrication of perovskite films towards commercialization of solar cells. Energy Environ. Sci..

[19] Yang F, Dong L, Jang D, Saparov B, Tam KC (2021). Low temperature processed fully printed efficient planar structure carbon electrode perovskite solar cells and modules. Adv. Energy Mater..

[20] Mei A, Li X, Liu L, Ku Z, Liu T (2014). A hole-conductor–free, fully printable mesoscopic perovskite solar cell with high stability. Science.

[21] Zhou H, Chen Q, Li G, Luo S, Song T-B (2014). Interface engineering of highly efficient perovskite solar cells. Science.

[22] Correa-Baena J-P, Saliba M, Buonassisi T, Grätzel M, Abate A (2017). Promises and challenges of perovskite solar cells. Science.

[23] Lin X, Wang Y, Su H, Qin Z, Zhang Z (2022). An in-situ formed tunneling layer enriches the options of anode for efficient and stable regular perovskite solar cells. Nano-Micro Lett..

[24] Massiot I, Cattoni A, Collin S (2020). Progress and prospects for ultrathin solar cells. Nat. Energy.

[25] Green MA, Dunlop ED, Hohl-Ebinger J, Yoshita M, Kopidakis N (2022). Solar cell efficiency tables (version 59). Prog. Photovolt..

[26] Japan Electrical Safety & Environment Technology Laboratories, Chinese Perovskite Tech Firm Wuxi Utmost Light Claims 20.5% ‘World Record’ Efficiency For Perovskite Solar Mini Module, Certified By Japan’s JET; Plans To Build Large Area Perovskite Solar Module Production Lines, http://taiyangnews.info/technology/20-5-world-record-efficiency-for-perovskite-solar-module/, 2021.

[27] Fu L, Li H, Wang L, Yin R, Li B (2020). Defect passivation strategies in perovskites for an enhanced photovoltaic performance. Energy Environ. Sci..

[28] Sha WEI, Ren X, Chen L, Choy WCH (2015). The efficiency limit of CH_3_NH_3_PbI_3_ perovskite solar cells. Appl. Phys. Lett..

[29] Draguta S, Christians JA, Morozov YV, Mucunzi A, Manser JS (2018). A quantitative and spatially resolved analysis of the performance-bottleneck in high efficiency, planar hybrid perovskite solar cells. Energy Environ. Sci..

[30] Ren X, Wang Z, Sha WEI, Choy WCH (2017). Exploring the way to approach the efficiency limit of perovskite solar cells by drift-diffusion model. ACS Photonics.

[31] Sha WEI, Zhang H, Wang ZS, Zhu HL, Ren X (2018). Quantifying efficiency loss of perovskite solar cells by a modified detailed balance model. Adv. Energy Mater..

[32] Zhao X, Park N-G (2015). Stability issues on perovskite solar cells. Photonics.

[33] Aftab A, Ahmad MI (2021). A review of stability and progress in tin halide perovskite solar cell. Sol. Energy.

[34] Wang R, Mujahid M, Duan Y, Wang ZK, Xue J (2019). A review of perovskites solar cell stability. Adv. Funct. Mater..

[35] Feng J, Zhu X, Yang Z, Zhang X, Niu J (2018). Record efficiency stable flexible perovskite solar cell using effective additive assistant strategy. Adv. Mater..

[36] Tavakoli MM, Yadav P, Tavakoli R, Kong J (2018). Surface engineering of TiO_2_ ETL for highly efficient and hysteresis-less planar perovskite solar cell (21.4%) with enhanced open-circuit voltage and stability. Adv. Energy Mater..

[37] Meng L, You J, Yang Y (2018). Addressing the stability issue of perovskite solar cells for commercial applications. Nat. Commun..

[38] Sultana N, Demarais NJ, Shevchenko D, Derrick PJ (2018). Laser desorption/ionization mass spectrometry of perovskite solar cells: identification of interface interactions and degradation reactions. Sol. RRL.

[39] Wei J, Wang Q, Huo J, Gao F, Gan Z (2021). Mechanisms and suppression of photoinduced degradation in perovskite solar cells. Adv. Energy Mater..

[40] Li N, Niu X, Chen Q, Zhou H (2020). Towards commercialization: the operational stability of perovskite solar cells. Chem. Soc. Rev..

[41] Wang J, Chen X, Jiang F, Luo Q, Zhang L (2018). Electrochemical corrosion of Ag electrode in the silver grid electrode-based flexible perovskite solar cells and the suppression method. Sol. RRL.

[42] Wang X, Rakstys K, Jack K, Jin H, Lai J (2021). Engineering fluorinated-cation containing inverted perovskite solar cells with an efficiency of >21% and improved stability towards humidity. Nat. Commun..

[43] Liu Z, Qiu L, Ono LK, He S, Hu Z (2020). A holistic approach to interface stabilization for efficient perovskite solar modules with over 2,000-hour operational stability. Nat. Energy.

[44] Yang S, Chen S, Mosconi E, Fang Y, Xiao X (2019). Stabilizing halide perovskite surfaces for solar cell operation with wide-bandgap lead oxysalts. Science.

[45] Hang P, Xie J, Kan C, Li B, Zhang Y (2021). Stabilizing fullerene for burn-in-free and stable perovskite solar cells under ultraviolet preconditioning and light soaking. Adv. Mater..

[46] Rong Y, Hu Y, Mei A, Tan H, Saidaminov MI (2018). Challenges for commercializing perovskite solar cells. Science.

[47] Ma X, Yang L, Shang X, Li M, Gao D (2021). Grain boundary defect passivation by in situ formed wide-bandgap lead sulfate for efficient and stable perovskite solar cells. Chem. Eng. J..

[48] Frost JM, Butler KT, Brivio F, Hendon CH, van Schilfgaarde M (2014). Atomistic origins of high-performance in hybrid halide perovskite solar cells. Nano Lett..

[49] Carrillo J, Guerrero A, Rahimnejad S, Almora O, Zarazua I (2016). Ionic reactivity at contacts and aging of methylammonium lead triiodide perovskite solar cells. Adv. Energy Mater..

[50] Christians JA, MirandaHerrera PA, Kamat PV (2015). Transformation of the excited state and photovoltaic efficiency of CH_3_NH_3_PbI_3_ perovskite upon controlled exposure to humidified air. J. Am. Chem. Soc..

[51] Leguy AMA, Hu Y, Campoy-Quiles M, Alonso MI, Weber OJ (2015). Reversible hydration of CH_3_NH_3_PbI_3_ in films, single crystals, and solar cells. Chem. Mater..

[52] Ren G, Han W, Zhang Q, Li Z, Deng Y (2022). Overcoming perovskite corrosion and de-doping through chemical binding of halogen bonds toward efficient and stable perovskite solar cells. Nano-Micro Lett..

[53] Tong G, Ono LK, Liu Y, Zhang H, Bu T (2021). Up-scalable fabrication of SnO_2_ with multifunctional interface for high performance perovskite solar modules. Nano-Micro Lett..

[54] Wu T, Qin Z, Wang Y, Wu Y, Chen W (2021). The main progress of perovskite solar cells in 2020–2021. Nano-Micro Lett..

[55] Li Z, Gao Y, Zhang Z, Xiong Q, Deng L (2021). cPCN-regulated SnO_2_ composites enables perovskite solar cell with efficiency beyond 23%. Nano-Micro Lett..

[56] Bryant D, Aristidou N, Pont S, Sanchez-Molina I, Chotchuangchutchaval T (2016). Light and oxygen induced degradation limits the operational stability of methylammonium lead triiodide perovskite solar cells. Energy Environ. Sci..

[57] Bryant D, Aristidou N, Pont S, Sanchez-Molina I, Chotchunangatchaval T (2016). Light and oxygen induced degradation limits the operational stability of methylammonium lead triiodide perovskite solar cells. Energy Environ. Sci..

[58] Chi W, Banerjee SK (2021). Achieving resistance against moisture and oxygen for perovskite solar cells with high efficiency and stability. Chem. Mater..

[59] Niu G, Guo X, Wang L (2015). Review of recent progress in chemical stability of perovskite solar cells. J. Mater. Chem. A.

[60] Li F, Liu M (2017). Recent efficient strategies for improving the moisture stability of perovskite solar cells. J. Mater. Chem. A.

[61] Jiang J, Wang Q, Jin Z, Zhang X, Lei J (2018). Polymer doping for high-efficiency perovskite solar cells with improved moisture stability. Adv. Energy Mater..

[62] Hwang I, Jeong I, Lee J, Ko MJ, Yong K (2015). Enhancing stability of perovskite solar cells to moisture by the facile hydrophobic passivation. ACS Appl. Mater. Interfaces.

[63] Yang J, Siempelkamp BD, Liu D, Kelly TL (2015). Investigation of CH_3_NH_3_PbI_3_ degradation rates and mechanisms in controlled humidity environments using in situ techniques. ACS Nano.

[64] Zhu J, Kim DH, Kim JD, Lee DG, Kim WB (2021). All-in-one Lewis base for enhanced precursor and device stability in highly efficient perovskite solar cells. ACS Energy Lett..

[65] Yu L, Guo T, Yuan H, Zhang Z, Deng Z (2021). Effective lewis base additive with S-donor for efficient and stable CsPbI_2_Br based perovskite solar cells. Chem. Eng. J..

[66] Xiang W, Liu S, Tress W (2021). A review on the stability of inorganic metal halide perovskites: challenges and opportunities for stable solar cells. Energy Environ. Sci..

[67] Xiong S, Yuan M, Yang J, Song J, Guo X (2019). Engineering of the back contact between PCBM and metal electrode for planar perovskite solar cells with enhanced efficiency and stability. Adv. Opt. Mater..

[68] Rivkin B, Fassl P, Sun Q, Taylor AD, Chen Z (2018). Effect of ion migration-induced electrode degradation on the operational stability of perovskite solar cells. ACS Omega.

[69] Yang Z, Babu BH, Wu S, Liu T, Fang S (2020). Review on practical interface engineering of perovskite solar cells: from efficiency to stability. Sol. RRL.

[70] Xu Y, Lin Z, Wei W, Hao Y, Liu S (2022). Recent progress of electrode materials for flexible perovskite solar cells. Nano-Micro Lett..

[71] Salim KMM, Masi S, Gualdrón-Reyes AF, Sánchez RS, Barea EM (2021). Boosting long-term stability of pure formamidinium perovskite solar cells by ambient air additive assisted fabrication. ACS Energy Lett..

[72] Zheng H, Xu X, Xu S, Liu G, Chen S (2019). The multiple effects of polyaniline additive to improve the efficiency and stability of perovskite solar cells. J. Mater. Chem. C.

[73] Xie L, Chen J, Vashishtha P, Zhao X, Shin GS (2019). Importance of functional groups in cross-linking methoxysilane additives for high-efficiency and stable perovskite solar cells. ACS Energy Lett..

[74] Park C, Ko H, Sin DH, Song KC, Cho K (2017). Organometal halide perovskite solar cells with improved thermal stability via grain boundary passivation using a molecular additive. Adv. Funct. Mater..

[75] Chen Y, Yang X, Liu P, Wang W, Ran R (2021). Improving moisture/thermal stability and efficiency of CH_3_NH_3_PbI_3_-based perovskite solar cells via gentle butyl acrylate additive strategy. Sol. RRL.

[76] Xu J, Cui J, Yang S, Han Y, Guo X (2021). Unraveling passivation mechanism of imidazolium-based ionic liquids on inorganic perovskite to achieve near-record-efficiency CsPbI_2_Br solar cells. Nano-Micro Lett..

[77] Zhao W, Xu J, He K, Cai Y, Han Y (2021). A special additive enables all cations and anions passivation for stable perovskite solar cells with efficiency over 23%. Nano-Micro Lett..

[78] Tang S, Deng Y, Zheng X, Bai Y, Fang Y (2017). Composition engineering in doctor-blading of perovskite solar cells. Adv. Energy Mater..

[79] Jiang Y, He X, Liu T, Zhao N, Qin M (2019). Intralayer a-site compositional engineering of ruddlesden–popper perovskites for thermostable and efficient solar cells. ACS Energy Lett..

[80] Ozturk T, Akman E, Shalan AE, Akin S (2021). Composition engineering of operationally stable CsPbI_2_Br perovskite solar cells with a record efficiency over 17%. Nano Energy.

[81] Jin X, Yang L, Wang X-F (2021). Efficient two-dimensional perovskite solar cells realized by incorporation of Ti_3_C_2_Tx MXene as nano-dopants. Nanomicro. Lett..

[82] Liu J, Pathak SK, Sakai N, Sheng R, Bai S (2016). Identification and mitigation of a critical interfacial instability in perovskite solar cells employing copper thiocyanate hole-transporter. Adv. Mater. Interfaces.

[83] Zhang J, Long J, Huang Z, Yang J, Li X (2021). Obstructing interfacial reaction between NiO_x_ and perovskite to enable efficient and stable inverted perovskite solar cells. Chem. Eng. J..

[84] Guarnera S, Abate A, Zhang W, Foster JM, Richardson G (2015). Improving the long-term stability of perovskite solar cells with a porous Al_2_O_3_ buffer layer. J. Phys. Chem. Lett..

[85] Capasso A, Matteocci F, Najafi L, Prato M, Buha J (2016). Few-layer MoS_2_ flakes as active buffer layer for stable perovskite solar cells. Adv. Energy Mater..

[86] Zheng X, Lei H, Yang G, Ke W, Chen Z (2017). Enhancing efficiency and stability of perovskite solar cells via a high mobility p-type PbS buffer layer. Nano Energy.

[87] Chatzimanolis K, Rogdakis K, Tsikritzis D, Tzoganakis N, Tountas M (2021). Inverted perovskite solar cells with enhanced lifetime and thermal stability enabled by a metallic tantalum disulfide buffer layer. Nanoscale Adv..

[88] Zhong H, Li W, Huang Y, Cao D, Zhang C (2022). All-inorganic perovskite solar cells with tetrabutylammonium acetate as the buffer layer between the SnO_2_ electron transport film and CsPbI_3_. ACS Appl. Mater. Interfaces.

[89] Dong W, Qiao W, Xiong S, Yang J, Wang X (2022). Surface passivation and energetic modification suppress nonradiative recombination in perovskite solar cells. Nano-Micro Lett..

[90] Zhu L, Chen C, Weng Y, Li F, Lou Q (2019). Enhancing the performance of inverted perovskite solar cells by inserting a ZnO:TIPD film between PCBM layer and Ag electrode. Sol. Energy Mater. Sol. Cells.

[91] Chang C-Y, Wang C-C (2020). Enhanced stability and performance of air-processed perovskite solar cells via defect passivation with a thiazole-bridged diketopyrrolopyrrole-based π-conjugated polymer. J. Mater. Chem. A.

[92] Chen J, Zuo L, Zhang Y, Lian X, Fu W (2018). High-performance thickness insensitive perovskite solar cells with enhanced moisture stability. Adv. Energy Mater..

[93] Han Y, Meyer S, Dkhissi Y, Weber K, Pringle JM (2015). Degradation observations of encapsulated planar CH_3_NH_3_PbI_3_ perovskite solar cells at high temperatures and humidity. J. Mater. Chem. A.

[94] Niu G, Li W, Meng F, Wang L, Dong H (2014). Study on the stability of CH3NH3PbI3 films and the effect of post-modification by aluminum oxide in all-solid-state hybrid solar cells. J. Mater. Chem. A.

[95] Wang Q, Chen B, Liu Y, Deng Y, Bai Y (2017). Scaling behavior of moisture-induced grain degradation in polycrystalline hybrid perovskite thin films. Energy Environ. Sci..

[96] Huang W, Manser JS, Kamat PV, Ptasinska S (2016). Evolution of chemical composition, morphology, and photovoltaic efficiency of CH_3_NH_3_PbI_3_ perovskite under ambient conditions. Chem. Mater..

[97] Ahn N, Kwak K, Jang MS, Yoon H, Lee BY (2016). Trapped charge-driven degradation of perovskite solar cells. Nat. Commun..

[98] Pearson AJ, Eperon GE, Hopkinson PE, Habisreutinger SN, Wang JT-W (2016). Oxygen degradation in mesoporous Al_2_O_3_/CH_3_NH_3_PbI_3-x_Cl_x_ perovskite solar cells: kinetics and mechanisms. Adv. Energy Mater..

[99] Zhang L, Sit PHL (2017). Ab initio study of the role of oxygen and excess electrons in the degradation of CH_3_NH_3_PbI_3_. J. Mater. Chem. A.

[100] Aristidou N, Sanchez-Molina I, Chotchuangchutchaval T, Brown M, Martinez L (2015). The role of oxygen in the degradation of methylammonium lead trihalide perovskite photoactive layers. Angew. Chem. Int. Ed..

[101] Aristidou N, Eames C, Sanchez-Molina I, Bu X, Kosco J (2017). Fast oxygen diffusion and iodide defects mediate oxygen-induced degradation of perovskite solar cells. Nat. Commun..

[102] Godding JSW, Ramadan AJ, Lin Y-H, Schutt K, Snaith HJ (2019). Oxidative passivation of metal halide perovskites. Joule.

[103] Lang F, Shargaieva O, Brus VV, Neitzert HC, Rappich J (2018). Influence of radiation on the properties and the stability of hybrid perovskites. Adv. Mater..

[104] Siegler TD, Dunlap-Shohl WA, Meng Y, Yang Y, Kau WF (2022). Water-accelerated photooxidation of CH_3_NH_3_PbI_3_ perovskite. J. Am. Chem. Soc..

[105] Ramadan AJ, Rochford LA, Fearn S, Snaith HJ (2017). Processing solvent-dependent electronic and structural properties of cesium lead triiodide thin films. J. Phys. Chem. Lett..

[106] Sun Q, Fassl P, Becker-Koch D, Bausch A, Rivkin B (2017). Role of microstructure in oxygen induced photodegradation of methylammonium lead triiodide perovskite films. Adv. Energy Mater..

[107] Ouyang Y, Li Y, Zhu P, Li Q, Gao Y (2019). Photo-oxidative degradation of methylammonium lead iodide perovskite: mechanism and protection. J. Mater. Chem. A.

[108] Li W, Zhang W, Van Reenen S, Sutton RJ, Fan J (2016). Enhanced UV-light stability of planar heterojunction perovskite solar cells with caesium bromide interface modification. Energy Environ. Sci..

[109] Leijtens T, Eperon GE, Pathak S, Abate A, Lee MM (2013). Overcoming ultraviolet light instability of sensitized TiO_2_ with meso-superstructured organometal tri-halide perovskite solar cells. Nat. Commun..

[110] Li Y, Xu X, Wang C, Ecker B, Yang J (2017). Light-induced degradation of CH_3_NH_3_PbI_3_ hybrid perovskite thin film. J. Phys. Chem. C.

[111] Song Z, Wang C, Phillips AB, Grice CR, Zhao D (2018). Probing the origins of photodegradation in organic–inorganic metal halide perovskites with time-resolved mass spectrometry. Sustain. Energy Fuels.

[112] Motti SG, Meggiolaro D, Barker AJ, Mosconi E, Perini CAR (2019). Controlling competing photochemical reactions stabilizes perovskite solar cells. Nat. Photonics.

[113] Azpiroz JM, Mosconi E, Bisquert J, De Angelis F (2015). Defect migration in methylammonium lead iodide and its role in perovskite solar cell operation. Energy Environ. Sci..

[114] Gottesman R, Haltzi E, Gouda L, Tirosh S, Bouhadana Y (2014). Extremely slow photoconductivity response of CH_3_NH_3_PbI_3_ perovskites suggesting structural changes under working conditions. J. Phys. Chem. Lett..

[115] You Y, Tian W, Min L, Cao F, Deng K (2020). TiO_2_/WO_3_ bilayer as electron transport layer for efficient planar perovskite solar cell with efficiency exceeding 20%. Adv. Mater. Interfaces.

[116] Byranvand MM, Kim T, Song S, Kang G, Ryu SU (2018). p-type CuI islands on TiO_2_ electron transport layer for a highly efficient planar-perovskite solar cell with negligible hysteresis. Adv. Energy Mater..

[117] Kim M, Choi I-W, Choi SJ, Song JW, Mo S-I (2021). Enhanced electrical properties of Li-salts doped mesoporous TiO_2_ in perovskite solar cells. Joule.

[118] Wang B, Yang J, Lu L, Xiao W, Wu H (2020). Interface engineering of air-stable n-doping fullerene-modified TiO_2_ electron transport layer for highly efficient and stable perovskite solar cells. Adv. Mater. Interfaces.

[119] Haque S, Mendes MJ, Sanchez-Sobrado O, Águas H, Fortunato E (2019). Photonic-structured TiO_2_ for high-efficiency, flexible and stable perovskite solar cells. Nano Energy.

[120] Leijtens T, Eperon GE, Pathak S, Abate A, Lee MM (2013). Overcoming ultraviolet light instability of sensitized TiO_2_ with meso-superstructured organometal tri-halide perovskite solar cells. Nat. Commun..

[121] Shahiduzzaman M, Hossain MI, Visal S, Kaneko T, Qarony W (2021). Spray pyrolyzed TiO_2_ embedded multi-layer front contact design for high-efficiency perovskite solar cells. Nano-Micro. Lett..

[122] Roiati V, Mosconi E, Listorti A, Colella S, Gigli G (2014). Stark effect in perovskite/TiO_2_ solar cells: evidence of local interfacial order. Nano Lett..

[123] Kerner RA, Rand BP (2017). Linking chemistry at the TiO_2_/CH_3_NH_3_PbI_3_ interface to current–voltage hysteresis. J. Phys. Chem. Lett..

[124] Ito S, Tanaka S, Manabe K, Nishino H (2014). Effects of surface blocking layer of Sb_2_S_3_ on nanocrystalline TiO_2_ for CH_3_NH_3_PbI_3_ perovskite solar cells. J. Phys. Chem. C.

[125] Zhang J, Pauporté T (2015). Effects of oxide contact layer on the preparation and properties of CH_3_NH_3_PbI_3_ for perovskite solar cell application. J. Phys. Chem. C.

[126] Tavakoli MM, Tavakoli R, Nourbakhsh Z, Waleed A, Virk US (2016). High efficiency and stable perovskite solar cell using ZnO/rGO QDs as an electron transfer layer. Adv. Mater. Interfaces.

[127] Si H, Liao Q, Zhang Z, Li Y, Yang X (2016). An innovative design of perovskite solar cells with Al_2_O_3_ inserting at ZnO/perovskite interface for improving the performance and stability. Nano Energy.

[128] Azmi R, Hwang S, Yin W, Kim T-W, Ahn TK (2018). High efficiency low-temperature processed perovskite solar cells integrated with alkali metal doped ZnO electron transport layers. ACS Energy Lett..

[129] Wang Z, Zhu X, Feng J, Wang C, Zhang C (2021). Antisolvent-and annealing-free deposition for highly stable efficient perovskite solar cells via modified ZnO. Adv. Sci..

[130] Idígoras J, Todinova A, Sánchez-Valencia JR, Barranco A, Borrás A (2016). The interaction between hybrid organic–inorganic halide perovskite and selective contacts in perovskite solar cells: an infrared spectroscopy study. Phys. Chem. Chem. Phys..

[131] Yang J, Siempelkamp BD, Mosconi E, De Angelis F, Kelly TL (2015). Origin of the thermal instability in CH_3_NH_3_PbI_3_ thin films deposited on ZnO. Chem. Mater..

[132] Cheng Y, Yang Q-D, Xiao J, Xue Q, Li H-W (2015). Decomposition of organometal halide perovskite films on zinc oxide nanoparticles. ACS Appl. Mater. Interfaces.

[133] Hao F, Stoumpos CC, Cao DH, Chang RPH, Kanatzidis MG (2014). Lead-free solid-state organic–inorganic halide perovskite solar cells. Nat. Photonics.

[134] Giustino F, Snaith HJ (2016). Toward lead-free perovskite solar cells. ACS Energy Lett..

[135] Li Z, Wang L, Liu R, Fan Y, Meng H (2019). Spontaneous interface ion exchange: passivating surface defects of perovskite solar cells with enhanced photovoltage. Adv. Energy Mater..

[136] You J, Meng L, Song T-B, Guo T-F, Yang Y (2016). Improved air stability of perovskite solar cells via solution-processed metal oxide transport layers. Nat. Nanotechnol..

[137] Lee H, Lee C (2018). Analysis of ion-diffusion-induced interface degradation in inverted perovskite solar cells via restoration of the Ag electrode. Adv. Energy Mater..

[138] Xu J, Buin A, Ip AH, Li W, Voznyy O (2015). Perovskite–fullerene hybrid materials suppress hysteresis in planar diodes. Nat. Commun..

[139] Wang Q, Chueh C-C, Eslamian M, Jen AK-Y (2016). Modulation of PEDOT: PSS pH for efficient inverted perovskite solar cells with reduced potential loss and enhanced stability. ACS Appl. Mater. Interfaces.

[140] Sun W, Li Y, Xiao Y, Zhao Z, Ye S (2017). An ammonia modified PEDOT: PSS for interfacial engineering in inverted planar perovskite solar cells. Org. Electron..

[141] Jiang Y, Yang S-C, Jeangros Q, Pisoni S, Moser T (2020). Mitigation of vacuum and illumination-induced degradation in perovskite solar cells by structure engineering. Joule.

[142] Li Y, Wang B, Liu T, Zeng Q, Cao D (2022). Interfacial engineering of PTAA/perovskites for improved crystallinity and hole extraction in inverted perovskite solar cells. ACS Appl. Mater. Interfaces.

[143] Liu T, Chen K, Hu Q, Zhu R, Gong Q (2016). Inverted perovskite solar cells: progresses and perspectives. Adv. Energy Mater..

[144] Wang H, Huang Z, Xiao S, Meng X, Xing Z (2021). An in situ bifacial passivation strategy for flexible perovskite solar module with mechanical robustness by roll-to-roll fabrication. J. Mater. Chem. A.

[145] Du M, Zhao S, Duan L, Cao Y, Wang H (2022). Surface redox engineering of vacuum-deposited NiOx for top-performance perovskite solar cells and modules. Joule.

[146] Chen W, Liu FZ, Feng XY, Djurišić AB, Chan WK (2017). Cesium doped NiOx as an efficient hole extraction layer for inverted planar perovskite solar cells. Adv. Energy Mater..

[147] Manders JR, Tsang SW, Hartel MJ, Lai TH, Chen S (2013). Solution-processed nickel oxide hole transport layers in high efficiency polymer photovoltaic cells. Adv. Funct. Mater..

[148] Boyd CC, Shallcross RC, Moot T, Kerner R, Bertoluzzi L (2020). Overcoming redox reactions at perovskite-nickel oxide interfaces to boost voltages in perovskite solar cells. Joule.

[149] Hou F, Su Z, Jin F, Yan X, Wang L (2015). Efficient and stable planar heterojunction perovskite solar cells with an MoO_3_/PEDOT: PSS hole transporting layer. Nanoscale.

[150] Jo JW, Seo MS, Park M, Kim JY, Park JS (2016). Improving performance and stability of flexible planar-heterojunction perovskite solar cells using polymeric hole-transport material. Adv. Funct. Mater..

[151] Meng Y, Hu Z, Ai N, Jiang Z, Wang J (2014). Improving the stability of bulk heterojunction solar cells by incorporating pH-neutral PEDOT: PSS as the hole transport layer. ACS Appl. Mater. Interfaces.

[152] Redondo-Obispo C, Ripolles TS, Cortijo-Campos S, Alvarez AL, Climent-Pascual E (2020). Enhanced stability and efficiency in inverted perovskite solar cells through graphene doping of PEDOT: PSS hole transport layer. Mater. Design..

[153] Liu C, Zhang L, Li Y, Zhou X, She S (2020). Highly stable and efficient perovskite solar cells with 22.0% efficiency based on inorganic–organic dopant-free double hole transporting layers. Adv. Funct. Mater..

[154] Kim S, Bae S, Lee S-W, Cho K, Lee KD (2017). Relationship between ion migration and interfacial degradation of CH_3_NH_3_PbI_3_ perovskite solar cells under thermal conditions. Sci. Rep..

[155] Wu T, Zhuang R, Zhao R, Zhao R, Zhu L (2021). Understanding the effects of fluorine substitution in lithium salt on photovoltaic properties and stability of perovskite solar cells. ACS Energy Lett..

[156] Liu Y, Hu Y, Zhang X, Zeng P, Li F (2020). Inhibited aggregation of lithium salt in spiro-OMeTAD toward highly efficient perovskite solar cells. Nano Energy.

[157] Li W, Dong H, Wang L, Li N, Guo X (2014). Montmorillonite as bifunctional buffer layer material for hybrid perovskite solar cells with protection from corrosion and retarding recombination. J. Mater. Chem. A.

[158] Yue Y, Salim N, Wu Y, Yang X, Islam A (2016). Enhanced stability of perovskite solar cells through corrosion-free pyridine derivatives in hole-transporting materials. Adv. Mater..

[159] Chen B-A, Lin J-T, Suen N-T, Tsao C-W, Chu T-C (2017). In situ identification of photo- and moisture-dependent phase evolution of perovskite solar cells. ACS Energy Lett..

[160] Wu Q, Xue C, Li Y, Zhou P, Liu W (2015). Kesterite Cu_2_ZnSnS_4_ as a low-cost inorganic hole-transporting material for high-efficiency perovskite solar cells. ACS Appl. Mater. Interfaces.

[161] Yang Y, Chen H, Zheng X, Meng X, Zhang T (2017). Ultrasound-spray deposition of multi-walled carbon nanotubes on NiO nanoparticles-embedded perovskite layers for high-performance carbon-based perovskite solar cells. Nano Energy.

[162] Liu C, Zhou X, Chen S, Zhao X, Dai S (2019). Hydrophobic Cu_2_O quantum dots enabled by surfactant modification as top hole-transport materials for efficient perovskite solar cells. Adv. Sci..

[163] Yang IS, Lee S, Choi J, Jung MT, Kim J (2019). Enhancement of open circuit voltage for CuSCN-based perovskite solar cells by controlling the perovskite/CuSCN interface with functional molecules. J. Mater. Chem. A.

[164] Lee B, Yun AJ, Kim J, Gil B, Shin B (2019). Aminosilane-modified CuGaO_2_ nanoparticles incorporated with CuSCN as a hole-transport layer for efficient and stable perovskite solar cells. Adv. Mater. Interfaces.

[165] Arora N, Dar MI, Hinderhofer A, Pellet N, Schreiber F (2017). Perovskite solar cells with CuSCN hole extraction layers yield stabilized efficiencies greater than 20%. Science.

[166] Sepalage GA, Meyer S, Pascoe AR, Scully AD, Bach U (2017). A facile deposition method for CuSCN: exploring the influence of CuSCN on JV hysteresis in planar perovskite solar cells. Nano Energy.

[167] Xu P, Liu J, Huang J, Yu F, Li C-H (2021). Interfacial engineering of CuSCN-based perovskite solar cells via PMMA interlayer toward enhanced efficiency and stability. New J. Chem..

[168] Kavan L, Živcová ZV, Hubík P, Arora N, Dar MI (2019). Electrochemical characterization of CuSCN hole-extracting thin films for perovskite photovoltaics. ACS Appl. Energy Mater..

[169] Kim H-S, Jang I-H, Ahn N, Choi M, Guerrero A (2015). Control of I-V hysteresis in CH_3_NH_3_PbI_3_ perovskite solar cell. J. Phys. Chem. Lett..

[170] Zhang T, Meng X, Bai Y, Xiao S, Hu C (2017). Profiling the organic cation-dependent degradation of organolead halide perovskite solar cells. J. Mater. Chem. A.

[171] Kato Y, Ono LK, Lee MV, Wang S, Raga SR (2015). Silver iodide formation in methyl ammonium lead iodide perovskite solar cells with silver top electrodes. Adv. Mater. Interfaces.

[172] Li J, Dong Q, Li N, Wang L (2017). Direct evidence of ion diffusion for the silver-electrode-induced thermal degradation of inverted perovskite solar cells. Adv. Energy Mater..

[173] Lee E, Ahn J, Kwon HC, Ma S, Kim K (2018). All-solution-processed silver nanowire window electrode-based flexible perovskite solar cells enabled with amorphous metal oxide protection. Adv. Energy Mater..

[174] Cheng Y, Liu X, Guan Z, Li M, Zeng Z (2021). Revealing the degradation and self-healing mechanisms in perovskite solar cells by sub-bandgap external quantum efficiency spectroscopy. Adv. Mater..

[175] Boyd CC, Cheacharoen R, Bush KA, Prasanna R, Leijtens T (2018). Barrier design to prevent metal-induced degradation and improve thermal stability in perovskite solar cells. ACS Energy Lett..

[176] Jeong J, Kim M, Seo J, Lu H, Ahlawat P (2021). Pseudo-halide anion engineering for α-FAPbI_3_ perovskite solar cells. Nature.

[177] Guerrero A, You J, Aranda C, Kang YS, Garcia-Belmonte G (2016). Interfacial degradation of planar lead halide perovskite solar cells. ACS Nano.

[178] Qin P, Wu T, Wang Z, Xiao L, Ma L (2020). Grain boundary and interface passivation with Core-Shell Au@CdS nanospheres for high-efficiency perovskite solar cells. Adv. Funct. Mater..

[179] Davis A, Tran T, Young D (1993). Solution chemistry of iodide leaching of gold. Hydrometallurgy.

[180] Liang L, Cai Y, Li X, Nazeeruddin MK, Gao P (2018). All that glitters is not gold: recent progress of alternative counter electrodes for perovskite solar cells. Nano Energy.

[181] Kaltenbrunner M, Adam G, Głowacki ED, Drack M, Schwödiauer R (2015). Flexible high power-per-weight perovskite solar cells with chromium oxide–metal contacts for improved stability in air. Nat. Mater..

[182] Cacovich S, Ciná L, Matteocci F, Divitini G, Midgley PA (2017). Gold and iodine diffusion in large area perovskite solar cells under illumination. Nanoscale.

[183] Domanski K, Correa-Baena J-P, Mine N, Nazeeruddin MK, Abate A (2016). Not all that glitters is gold: metal-migration-induced degradation in perovskite solar cells. ACS Nano.

[184] Shlenskaya NN, Belich NA, Grätzel M, Goodilin EA, Tarasov AB (2018). Light-induced reactivity of gold and hybrid perovskite as a new possible degradation mechanism in perovskite solar cells. J. Mater. Chem. A.

[185] Kerner RA, Schulz P, Christians JA, Dunfield SP, Dou B (2019). Reactions at noble metal contacts with methylammonium lead triiodide perovskites: role of underpotential deposition and electrochemistry. APL Mater..

[186] Xu Y, Tian Y, Hou M, Wu Y, Ding Y (2020). Performance promotion through dual-interface engineering of CuSCN layers in planar perovskite solar cells. J. Phys. Chem. C.

[187] Yang Y, Hoang MT, Yao D, Pham ND, Tiong VT (2020). Spiro-OMeTAD or CuSCN as a preferable hole transport material for carbon-based planar perovskite solar cells. J. Mater. Chem. A.

[188] Jørgensen M, Norrman K, Krebs FC (2008). Stability/degradation of polymer solar cells. Sol. Energy Mater. Sol. Cells.

[189] Guo S, Sun X, Ding C, Huang R, Tan M (2020). Non-uniform chemical corrosion of metal electrode of p–i–n type of perovskite solar cells caused by the diffusion of CH_3_NH_3_I. Energy Technol..

[190] Zhao L, Kerner RA, Xiao Z, Lin YL, Lee KM (2016). Redox chemistry dominates the degradation and decomposition of metal halide perovskite optoelectronic devices. ACS Energy Lett..

[191] Lin Y-H, Sakai N, Da P, Wu J, Sansom Harry C (2020). A piperidinium salt stabilizes efficient metal-halide perovskite solar cells. Science.

[192] Kim M, Motti SG, Sorrentino R, Petrozza A (2018). Enhanced solar cell stability by hygroscopic polymer passivation of metal halide perovskite thin film. Energy Environ. Sci..

[193] Wang Q, Dong Q, Li T, Gruverman A, Huang J (2016). Thin insulating tunneling contacts for efficient and water-resistant perovskite solar cells. Adv. Mater..

[194] Wu Z, Song T, Sun B (2017). Carbon-based materials used for perovskite solar cells. ChemNanoMat.

[195] Gao L, Spanopoulos I, Ke W, Huang S, Hadar I (2019). Improved environmental stability and solar cell efficiency of (MA, FA) PbI_3_ perovskite using a wide-band-gap 1D thiazolium lead iodide capping layer strategy. ACS Energy Lett..

[196] Hou Y, Du X, Scheiner S, McMeekin DP, Wang Z (2017). A generic interface to reduce the efficiency-stability-cost gap of perovskite solar cells. Science.

[197] Kim J, Lee Y, Gil B, Yun AJ, Kim J (2020). A Cu_2_O–CuSCN nanocomposite as a hole-transport material of perovskite solar cells for enhanced carrier transport and suppressed interfacial degradation. ACS Appl. Energy Mater..

[198] Mali SS, Patil JV, Kim H, Luque R, Hong CK (2019). Highly efficient thermally stable perovskite solar cells via Cs:NiO_x_/CuSCN double-inorganic hole extraction layer interface engineering. Mater. Today.

[199] Cao J, Wu B, Chen R, Wu Y, Hui Y (2018). Efficient, hysteresis-free, and stable perovskite solar cells with ZnO as electron-transport layer: effect of surface passivation. Adv. Mater..

[200] Azmi R, Lee C-L, Jung IH, Jang S-Y (2018). Simultaneous improvement in efficiency and stability of low-temperature-processed perovskite solar cells by interfacial control. Adv. Energy Mater..

[201] Qiu W, Buffière M, Brammertz G, Paetzold UW, Froyen L (2015). High efficiency perovskite solar cells using a PCBM/ZnO double electron transport layer and a short air-aging step. Org. Electron..

[202] Zheng S, Li W, Su T, Xie F, Chen J (2018). Metal oxide cro_x_ as a promising bilayer electron transport material for enhancing the performance stability of planar perovskite solar cells. Sol. RRL.

[203] Jiang Z, Chen X, Lin X, Jia X, Wang J (2016). Amazing stable open-circuit voltage in perovskite solar cells using AgAl alloy electrode. Sol. Energy Mater. Sol. Cells.

[204] Bi E, Chen H, Xie F, Wu Y, Chen W (2017). Diffusion engineering of ions and charge carriers for stable efficient perovskite solar cells. Nat. Commun..

[205] Lee J, Singh S, Kim S, Baik S (2020). Graphene interfacial diffusion barrier between CuSCN and Au layers for stable perovskite solar cells. Carbon.

[206] Jiang Q, Tong J, Xian Y, Kerner RA, Dunfield SP (2022). Surface reaction for efficient and stable inverted perovskite solar cells. Nature.

[207] Li X, Zhang W, Guo X, Lu C, Wei J (2022). Constructing heterojunctions by surface sulfidation for efficient inverted perovskite solar cells. Science.

[208] Zhang F, Zhu K (2020). Additive engineering for efficient and stable perovskite solar cells. Adv. Energy Mater..

[209] Bi D, Gao P, Scopelliti R, Oveisi E, Luo J (2016). High-performance perovskite solar cells with enhanced environmental stability based on amphiphile-modified CH_3_NH_3_PbI_3_. Adv. Mater..

[210] Zhao Y, Wei J, Li H, Yan Y, Zhou W (2016). A polymer scaffold for self-healing perovskite solar cells. Nat. Commun..

[211] Li X, Zhang W, Zhang W, Wang H-Q, Fang J (2019). Spontaneous grain polymerization for efficient and stable perovskite solar cells. Nano Energy.

[212] Zuo L, Guo H, deQuilettes DW, Jariwala S, deMarco N (2017). Polymer-modified halide perovskite films for efficient and stable planar heterojunction solar cells. Sci. Adv..

[213] Li X, Zhang W, Wang Y-C, Zhang W, Wang H-Q (2018). In-situ cross-linking strategy for efficient and operationally stable methylammoniun lead iodide solar cells. Nat. Commun..

[214] Bai S, Da P, Li C, Wang Z, Yuan Z (2019). Planar perovskite solar cells with long-term stability using ionic liquid additives. Nature.

[215] Gao L, Huang S, Chen L, Li X, Ding B (2018). Excellent stability of perovskite solar cells by passivation engineering. Sol. RRL.

[216] Kim GY, Senocrate A, Yang T-Y, Gregori G, Grätzel M (2018). Large tunable photoeffect on ion conduction in halide perovskites and implications for photodecomposition. Nat. Mater..

[217] Lou Q, Li H, Huang Q, Shen Z, Li F (2021). Multifunctional CNT: TiO_2_ additives in spiro-OMeTAD layer for highly efficient and stable perovskite solar cells. EcoMat.

[218] Luo Q, Zhang Y, Liu C, Li J, Wang N (2015). Iodide-reduced graphene oxide with dopant-free spiro-OMeTAD for ambient stable and high-efficiency perovskite solar cells. J. Mater. Chem. A.

[219] Han Y, Zhang G, Xie H, Kong T, Li Y (2022). Azide additive acting as a powerful locker for Li^+^ and TBP in spiro-OMeTAD toward highly efficient and stable perovskite solar cells. Nano Energy.

[220] Shao Y, Fang Y, Li T, Wang Q, Dong Q (2016). Grain boundary dominated ion migration in polycrystalline organic–inorganic halide perovskite films. Energy Environ. Sci..

[221] Wang R, Xue J, Meng L, Lee J-W, Zhao Z (2019). Caffeine improves the performance and thermal stability of perovskite solar cells. Joule.

[222] Liu L, Huang S, Lu Y, Liu P, Zhao Y (2018). Grain-boundary “patches” by in situ conversion to enhance perovskite solar cells stability. Adv. Mater..

[223] Li X, Ke S, Feng X, Zhao X, Zhang W (2021). Enhancing the stability of perovskite solar cells through cross-linkable and hydrogen bonding multifunctional additives. J. Mater. Chem. A.

[224] Ma Y, Cheng Y, Xu X, Li M, Zhang C (2021). Suppressing ion migration across perovskite grain boundaries by polymer additives. Adv. Funct. Mater..

